# Deletion of glutaredoxin promotes oxidative tolerance and intracellular infection in *Listeria monocytogenes*

**DOI:** 10.1080/21505594.2019.1685640

**Published:** 2019-11-02

**Authors:** Jing Sun, Yi Hang, Yue Han, Xian Zhang, Li Gan, Chang Cai, Zhongwei Chen, Yang Yang, Quanjiang Song, Chunyan Shao, Yongchun Yang, Yingshan Zhou, Xiaodu Wang, Changyong Cheng, Houhui Song

**Affiliations:** a Key Laboratory of Applied Technology on Green-Eco-Healthy Animal Husbandry of Zhejiang Province, China-Australian Joint Laboratory for Animal Health Big Data Analytics, Zhejiang Provincial Engineering Laboratory for Animal Health Inspection & Internet Technology, College of Animal Science and Technology & College of Veterinary Medicine of Zhejiang A&F University, Hangzhou, P. R. China; b School of Veterinary and Life Sciences, Murdoch University, Murdoch, Australia

**Keywords:** *Listeria monocytogenes*, glutaredoxin, oxidoreduction, oxidative tolerance, intracellular infection

## Abstract

Thiol-disulfide glutaredoxin systems of bacterial cytoplasm favor reducing conditions for the correct disulfide bonding of functional proteins, and therefore were employed by bacteria to defend against oxidative stress. *Listeria monocytogenes* has been shown to encode a putative glutaredoxin, Grx (encoded by *lmo2344*), while the underlying roles remain unknown. Here we suggest an unexpected role of *L. monocytogenes* Grx in oxidative tolerance and intracellular infection. The recombinant Grx was able to efficiently catalyze the thiol-disulfide oxidoreduction of insulin in the presence of DTT as an election donor. Unexpectedly, the deletion of *grx* resulted in a remarkably increased tolerance and survival ability of this bacteria when exposed to various oxidizing agents, including diamide, and copper and cadmium ions. Furthermore, loss of *grx* significantly promoted bacterial invasion and proliferation in human epithelial Caco-2 cells and murine macrophages, as well as a notably increasing invasion but not cell-to-cell spread in the murine fibroblasts L929 cells. More importantly, *L. monocytogenes* lacking the glutaredoxin exhibited more efficient proliferation and recovery in the spleens and livers of the infected mice, and hence became more virulent by upregulating the virulence factors, InlA and InlB. In summary, we here for the first time demonstrated that *L. monocytogenes* glutaredoxin plays a counterintuitive role in bacterial oxidative resistance and intracellular infection, which is the first report to provide valuable evidence for the role of glutaredoxins in bacterial infection, and more importantly suggests a favorable model to illustrate the functional diversity of bacterial Grx systems during environmental adaption and host infection.

## Introduction

The bacterial cytoplasm is a reducing environment and protein thiols are maintained in their reduced state by low molecular weight (LMW) thiol-redox buffers and enzymatic thiol-disulfide oxidoreductases, including the thioredoxin (Trx) and glutaredoxin (Grx) systems [1,2]. In their natural environment or during host infection, bacteria will encounter different oxidative stresses. The exposure of cells to oxidative conditions damage cellular components, including DNA, membrane lipids and proteins. Therefore, most organisms produce catalases, peroxiredoxins, and oxidoreductases that can react with harmful oxidants and convert them to harmless products before they cause damage to cellular components [3,4], with no exception for the intracellular foodborne pathogen *Listeria monocytogenes. L. monocytogenes* is a gram-positive facultative bacterial pathogen that can cause serious infections leading to high mortality in the immunocompromised individuals and pregnant women [5,6]. This organism is well-adapted to various physiological environments, employing various strategies to counteract hostile acidity, osmolarity, oxygen tension, and other stress conditions present in the environment and within the vacuolar compartment of phagocytic cells [7,8].

The thioredoxin super-family members, Trx and Grx systems can catalyze biological thiol-disulfide exchange reactions and play vital roles in a wide spectrum of cellular functions including redox sensing, cell signaling, cellular redox homeostasis, oxidative protein folding, and regulation of protein thiol function. In numerous bacterial species, such as *Staphylococcus aureus, Escherichia coli, Bacillus subtilis, Rhodobacter sphaeroides*, and *Brevibacillus choshinensis*, the presence of a functional Trx-dependent reduction pathway is required to maintain the intracellular thiol–disulfide homeostasis and for the protection of cells against oxidative stress. Thioredoxin is ubiquitous in bacteria, whereas the Grx antioxidant system is lacking in some specific bacteria [9]. Similar to the Trx system, the gluraredoxin system is composed of NADPH, glutathione reductase (GR), glutathione (GSH), and glutaredoxins [10]. All glutaredoxins share some structural similarity to Trxs. Most Grxs contain a CXXC catalytic motif (CPYC) and are therefore referred to as dithiol enzymes, whereas the rest have a CXXS catalytic motif (CGFS) and are referred to as monothiol enzymes [11,12]. Unlike the better characterized Trx system, much less is known about the role of Grx in bacteria. However, *E. coli* has been extensively studied and is a modern bacterium with Trx and Grx systems. The presence of the Grx system in *E. coli* provides a strong backup for the Trx system to participate in the antioxidant process by deglutathionylation as in mammalian cells. In *E. coli*, the dithiol Grx1, Grx2 and Grx3 enzymes are involved in the reduction of oxidized Cys in substrate proteins, whereas the monothiol Grx4 enzyme contributes to the assembly of iron–sulfur clusters [13]. Moreover, *E. coli* mutants that lack Grx3, but not mutants that lack Grx1 or Grx2, are killed more efficiently by oxidizing agents [14]. In addition, a recent study provided further evidence that *grxB* in *Cronobacter sakazakii* contributes to acid tolerance and plays a major positive role in cell surface hydrophobicity, agglutination, and bioﬁlm formation. To date, the biological functions of Grx in responses to oxidative stresses and host infection in gram-positive bacteria have not been investigated, which thus prompted us to elucidate the roles of glutaredoxin in the foodborne pathogen *L. monocytogenes*.

*L. monocytogenes lmo2344* is annotated as a putative glutaredoxin in the GenBank database. Homologs of the oxidoreductase system related genes have been identiﬁed in the sequenced genome of *L. monocytogenes* EGD-e *via* in silico analysis. Based on a pattern search for the CXXC motif, the characteristic structure of the thioredoxin super-family members in the EGD-e genome, only Lmo2344 contains a glutaredoxin domain, which might be suggested to function as a putative glutaredoxin [15]. However, none of the components of the Grx system from *L. monocytogenes* has been characterized to date. In the current study, we aimed to elucidate the molecular functions and underlying mechanisms of the *L. monocytogenes* Grx system, with a view to determine whether it contributes to biological processes related to bacterial survival and infection. Our novel findings demonstrate that *L. monocytogenes* glutaredoxin plays a counterintuitive role in bacterial oxidative resistance and intracellular infection, lack of Grx remarkably rendered *L. monocytogenes* more tolerant to the oxidizing environment, and more importantly, rendered this pathogen more efficient to invade and cell-to-cell spread during infection on host cells and mice model. The results from this study are the first report to provide valuable evidence for clarifying the pathways associated with the diverse roles of glutaredoxins from foodborne pathogens in improving survival in the external environment, and more importantly, successfully establishing infection within the host.

## Material and methods

### Bacterial strains, plasmids, primers, and culture conditions

*L. monocytogenes* EGD-e was used as the wild-type strain. *E. coli* DH5α was employed for cloning experiments and as the host strain for plasmids pET30a(+) (Merck), pIMK2 and pKSV7. *E. coli* Rosetta (DE3) was used for prokaryotic protein expression. *L. monocytogenes* strains were cultured in brain-heart infusion (BHI) medium (Oxoid). *E. coli* strains were grown at 37°C in Luria-Bertani broth (LB) (Oxoid). Stock solutions of ampicillin (50 mg/ml), erythromycin (50 mg/ml), kanamycin (50 mg/ml), or chloramphenicol (10 mg/ml) were added to medium where appropriate. All chemicals were obtained from Sangon Biotech, Merck or Sigma-Aldrich and were of the highest purity available. All primers used in this study are listed in Table S1 in Supplementary Material.

### Bioinformatics analysis

The amino acid sequences of putative glutaredoxins from *L. monocytogenes* EGD-e and its homologs from other microbial species were obtained from the National Center for Biotechnology Information database (NCBI GenBank). The sequences were aligned with the MUSCLE method by using CLC Sequence software. The phylogenetic tree was constructed with the Neighbor-Joining (NJ) method using 100 bootstrap replicates [16].

### Over-expression and purification of His-tagged Grx from E. coli

Recombinant proteins used here were expressed in the form of fusion proteins with the C-terminal His-tag by using pET30a(+) as the expression vector. Rosetta (DE3) was employed as the expression host. The full-length ORF of *lmo2344* gene from EGD-e genome was amplified, inserted into the expression vector, and finally transformed into Rosetta competent cells. Cells harboring recombinant plasmids were grown in 500 mL LB medium supplemented with 50 μg/mL kanamycin at 37°C until the cultures reached 0.8–1.0 at OD_600_ _nm_. Isopropyl β-D-1-thiogalactopyranoside (IPTG) was then added to a final concentration of 1 mM to induce expression of the recombinant proteins for additional 4 hours under the optimized conditions. The His-tagged fusion recombinant proteins were purified using the nickel-chelated affinity column chromatography, and concentrations of protein determined by using the BCA method.

### Site-directed mutagenesis

The site-directed mutants of Grx (Grx_CPYC_ and Grx_CGFS_) were generated using the original expressing plasmid, pET30a-Grx, and the QuikChange Site-Directed Mutagenesis kit (Agilent Technologies) with the primer pairs (Table S1 in Supplementary Material). Template DNA was removed via digestion with DpnI (TOYOBO) for 2 h at 37°C. All mutant constructs were sequenced to ensure that only the desired mutations had been incorporated correctly. The corresponding mutant proteins were expressed and purified as described above.

### In vitro oxidoreductase activity assays

The ability of Grx to catalyze reduction of human insulin (sigma) in the presence of DTT was measured as described previously [17,18]. The activity has been used to determine whether the glutaredoxin and its sites mutants were able to reduce disulfide-bridged insulin, using the thioredoxin from *L. monocytogenes* as a positive control [8]. The rate of insulin disulfide reduction was monitored spectrophotometrically following the turbidity at 650 nm. The reaction mixture contained 10 mM potassium phosphate buffer (pH 7.0), 0.15 mM insulin, 2 mM EDTA, and 10–100 μM of each redoxin. The reaction was started by adding 1 mM DTT as reductant. The non-enzymatic reduction of insulin by DTT was used as a negative control.

### In-frame deletion of L. monocytogenes gene

The temperature-sensitive pKSV7 shuttle vector was employed for creating gene mutations from *L. monocytogenes* using the homologous recombination strategy. Specifically, the DNA fragments containing homologous arms upstream and downstream of *lmo2344* were obtained by PCR amplification using the SOE primer pairs (Table S1 in Supplementary Material). The obtained fragment was cloned into the pKSV7 and then electroporated into EGD-e competent cells. Transformants were grown at a non-permissive temperature (42°C) in BHI broth containing 10 μg/mL chloramphenicol to promote chromosomal integration, and after that the recombinants were then passaged successively in BHI broth without antibiotics at a permissive temperature (30°C) for enabling plasmid excision and curing. Finally, the recombinant colonies were identified as chloramphenicol-sensitive ones, and the directed mutations further validated by DNA sequencing.

### Complementation of gene deletion mutant

To complement the *L. monocytogenes* Δ*grx* mutant, we constructed a complemented strain by using the integrative plasmid pIMK2. The complete open reading frame (ORF) of *grx* along with its native promoter (promoter of the whole operon) was amplified using the primer pairs (Table S1 in Supplementary Material) and cloned into pIMK2 plasmid. The resulting plasmid was then electroporated into *L. monocytogenes* Δ*grx* strain. Regenerated cells were plated on BHI agar containing kanamycin (50 μg/mL). The complemented strain was designated as CΔ*grx*.

### In vitro growth of L. monocytogenes mutants in BHI broth and agar medium

*L. monocytogenes* wild-type and gene deletion mutant strains were grown overnight at 37°C in BHI broth with shaking. Cultures were collected by centrifugation at 5,000 × g at 4°C, washed once in phosphate-buffered saline (PBS) (10 mM, pH 7.4) and initial OD_600_ _nm_ adjusted to 1.0 (∼10^9^ cfu ml^−1^). Bacteria were diluted (1:100) in fresh BHI broth, and incubated at 37°C for 12 h. For broth growth, the kinetic growth was conducted on the 96-wells and the bacterial OD measured at 1-hour interval. For plate growth, the bacteria were enumerated by plating serial dilutions of homogenates on BHI agar plates at 4 hours interval.

### Bacterial morphology

*L. monocytogenes* wild-type and gene deletion mutant strains were grown on the BHI agar plates for 12 hours, and the bacterial colony morphology was observed by using a stereo-microscope. The single bacterial morphology for each strain was observed by the transmission electron microscopy (TEM). The size of the single bacterial colony was measured of 100 bacteria per condition.

### Oxidative stress sensitivity assays

For oxidative stress, H_2_O_2_ was used as a direct oxidant and diamide as a thiol-specific oxidizing agent while the divalent metal ions such as copper and cadmium were used as the redox-active stress. *L. monocytogenes* wild-type and isogenic *grx* deletion mutant strains were grown overnight in BHI broth, and then diluted to OD_600_ _nm_ of 1.0 (∼10^9^ cfu ml^−1^) with 10 mM PBS (pH 7.4). Bacterial suspension was serially diluted 10-fold, and 10 μL of each dilution spotted onto BHI agar plates containing various concentrations of H_2_O_2_ (10–20 mM), diamide (1–2 mM), cadmium chloride (0.25–1 mM) or copper chloride (0.25–1 mM). Following incubation at 37°C for 24–48 hours, colony growth on each plate was assessed and imaged. All spot titre assays were performed in duplicate to verify the results.

### Proliferation of L. monocytogenes in J774A.1 macrophages

Intracellular growth was performed accordingly on the macrophages. Stationary *L. monocytogenes* were washed and re-suspended in 10 mM PBS (pH 7.4). Monolayers of J774 cells cultured in DMEM containing 10% fetal bovine serum (FBS, GE Healthcare Hyclone) were infected with bacteria for 30 min with MOI at 0.05 and incubated in DMEM containing gentamicin at 50 μg/mL for additional 30 min to kill extracellular bacteria. Then infected cells incubated in DMEM containing gentamicin at 5 μg/mL and 10% FBS, were lysed by adding 300 μL trypsin and 700 μL ice-cold sterile distilled water at 2, 6, or 12 hours post infection. The lysates were 10-fold diluted for enumeration of viable bacteria on BHI agar plates as mentioned above.

### Changes in transcription levels of virulence associated genes in the absence of grx

To further establish, whether Grx is involved in bacterial virulence, qRT-PCR was employed to analyze the changes in transcripts of the major virulence-associated genes, *prfA, hly, plcA, plcB, mpl, inlA* and *inlB*. Bacteria grown to the stationary phase were exposed to the oxidative stress (4 mM diamide) for 1 h. Total RNA was extracted using the Column Bacterial total RNA Puriﬁcation Kit (Sangon), according to the manufacturer’s instructions, genomic DNA removed using DNase I (TaKaRa) and cDNA synthesized with reverse transcriptase (TOYOBO). Real-time quantitative PCR was performed in a 20 µL reaction volume containing 200 ng cDNA, 10 µL SYBR quantitative PCR mix (TOYOBO), and 0.5 µL gene speciﬁc primers (200 nM, Supplementary Table S1) to measure the transcriptional levels of specific genes using the Mx3000P PCR detection system (Agilent). The housekeeping gene, 16SrRNA, was used as an internal control for normalization. Relative transcription levels were quantiﬁed using the 2^−ΔΔCT^ method and shown as relative fold changes. Triplicate assays were performed for each gene.

## Changes in expression levels of InlB in the absence of grx

To validate the regulation of Grx on InlB, western blot was employed to analyze the changes in expression of InlB in the presence or absence of Grx. For whole cell lysates: Bacterial pellet was resuspended in 1 mL extraction solution (2% Triton X-100, 1% SDS, 100 mM NaCl, 10 mM Tris-HCl, 1 mM EDTA, pH 8.0) and lysed by using the homogenizer (Bertin) at 6,000 rpm for 30 s with intermittent cooling for 30 s and then centrifuged at 12,000 g for 15 min. The pellet was discarded, and supernatant retained as the whole cell extract. For cell wall surface proteins: Bacterial pellets were resuspended in approximately 0.5% of the original culture volume of 10 mM PBS containing 2% (w/v) SDS for 30 min at 37°C with gentle shaking. Bacterial suspensions were centrifuged at 12,000 g for 10 min, the supernatant contained the extracted cell wall proteins was applied to an 0.22 μm filter and the filtrate was ready for use. Protein samples were separated using 12% SDS-PAGE and immunoblotted with α-InlB or α-GAPDH antisera (prepared in this study). GAPDH was employed as an internal control for the whole cell lysate.

### Adhesion and invasion of L. monocytogenes in Caco-2 cells

Bacterial invasion and proliferation inside human intestinal epithelial Caco-2 cells was performed according to the previous study [19]. Briefly, overnight grown *L. monocytogenes* cells were washed and re-suspended in 10 mM PBS (pH 7.4). Monolayers of Caco-2 cells, cultured in DMEM containing 20% FBS were infected with bacteria for 30 min with MOI at 10. For adhesion, cells were lysed after being washed three times with PBS. For invasion, cells were infected with bacteria for 90 min. Then cells were incubated in DMEM containing gentamicin at 50 μg/mL for additional 90 min to kill extracellular bacteria. At this time, cells were washed for estimation of invasion and lysates were 10-fold diluted for enumeration of viable bacteria on BHI agar plates. The adhesion or invasion efficiency was calculated by dividing the number of CFU that adhered or invaded the cells by the total infected number of bacteria.

### Intracellular multiplication assay in Caco-2 cells

Intracellular multiplication assay was conducted according to our previous work [16]. The Caco-2 cells were infected with *Listeria* at MOI of 10 at 37°C for 1.5 h. Extracellular bacteria were then killed with 50 μg/mL gentamycin for 1.5 h, and incubated for an additional 6 h. Cells were washed gently with 10 mM PBS (pH 7.4), fixed with 4% paraformaldehyde and then permeabilized with 0.5% Triton X-100. The bacterial cells were stained with polyclonal antibodies to *L. monocytogenes* for 1 h at 37°C, washed twice with PBS, and probed with Alexa Fluor 488-conjugated donkey anti-rabbit antibody (Santa Cruz) for 1 h at 37°C. F-actin was then stained with phalloidin-Alexa Fluor 568 (Thermo Fisher Scientific). DAPI (4′,6-diamidino-2-phenylindole) (Thermo Fisher Scientific) was used to stain the nuclei. Actin tails recruited by the bacteria were visualized under a ZEISS LSM510 confocal microscope (Zeiss Germany).

### Plaque assay in L929 fibroblast cells

The plaque assay was performed as previously described. Briefly, monolayers of mouse L929 fibroblast cells were maintained in high-glucose DMEM medium (Thermo Fisher Scientific) plus FBS (Hyclone) and 2 mM l-glutamine. Cells were plated at 1 × 10^6^ cells per well in a 6-well dish and infected at an MOI of 2.5 or 5 with *L. monocytogenes* under 37°C with 5% CO2 for 1 h. Extracellular bacteria were killed with 100 μg/mL gentamicin, and cells washed three times with 10 mM PBS (pH 7.4) and then overlaid with 3 mL of medium plus 0.7% agarose and 10 μg/mL gentamicin. Cells were fixed with paraformaldehyde (4% in PBS) for 20 min and stained with crystal violet after 72 hours infection for plaques observation.

### Virulence in the mouse model

*L. monocytogenes* wild-type and mutant strains were tested for the ability to recover from mice organs (livers and spleens). Briefly, The ICR mice (female, 18–22 g) (8 mice each group) were inoculated intraperitoneally with 10^6^ CFU of each strain. At 24 and 48 h post-infection, mice were sacrificed, and livers and spleens removed and individually homogenized in 10 mM PBS (pH 7.4). Surviving bacterial cells were enumerated by plating serial dilutions of homogenates on BHI agar plates.

### Statistical analysis

All experiments were repeated at least three times. Data were analyzed using the two-tailed homoscedastic Student’s t-test. Differences with *P*-values <0.05 were considered as statistically significant.

## Results

### L. monocytogenes Grx is capable to catalyze the reduction of insulin

**Figure 1.**
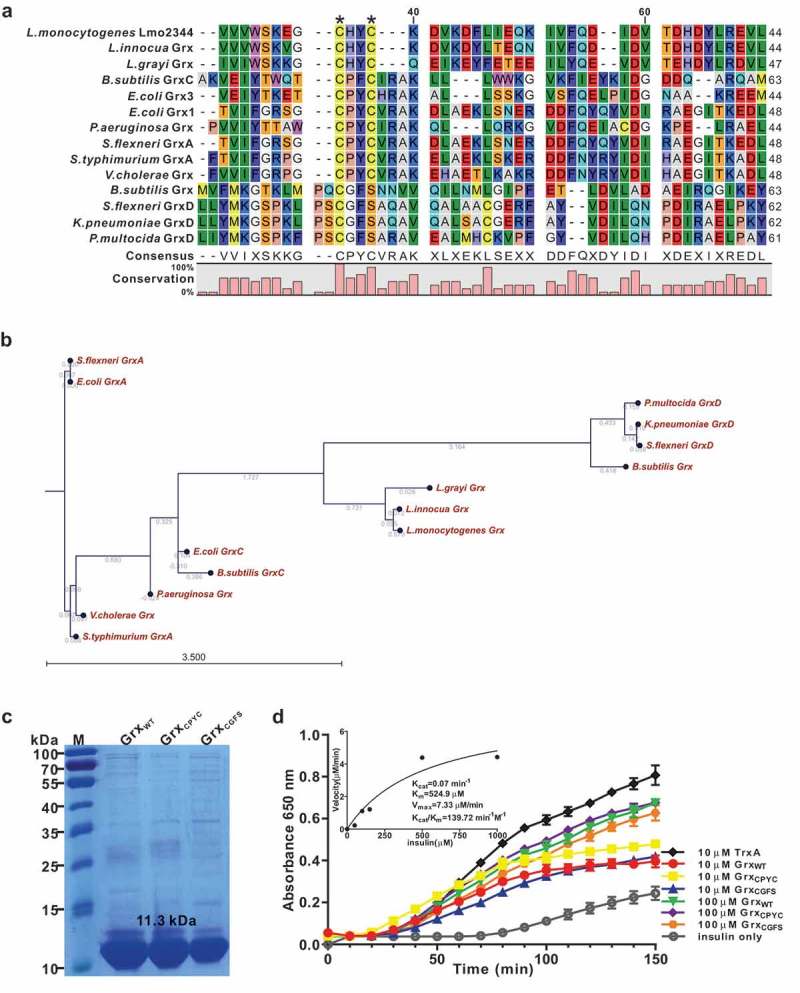
*L. monocytogenes* encodes a putative glutaredoxin, Grx that can catalyze reduction of insulin in the presence of DTT as an electron donor. (a) Amino acid sequence alignment of *L. monocytogenes* putative glutaredoxin (*lmo2344*) against homologues from *L. innocua, L. grayi, B. subtilis, P. aeruginosa, S. flexneri, S.typhimurium, V.cholerae, K.pneumoniae*,and *P. multocida*. The conserved CXXC catalytic motifs are denoted with asterisks. (b) Phylogenetic tree of *L. monocytogenes* putative glutaredoxin and homologs from the above bacterial species. The tree was constructed with the Neighbor-Joining (NJ) program and a bootstrap test of 100 replicates used to estimate the confidence of branching patterns, where the numbers on internal nodes represent the support values. (c) SDS-PAGE analysis of the purified histidine-tagged recombinant Grx and its site-directed mutants (Grx_CPYC_ and Grx_CGFS_) overexpressed in *E. coli*. (d) *In vitro* insulin disulfide reductase activity of recombinant Grx and its site-directed mutants (Grx_CPYC_ and Grx_CGFS_). The rate of insulin reduction was assessed by measuring the turbidity at 650 nm in reaction assays containing 1 mM DTT in the presence of 10–100 μM redoxins. The inset shows the Michaelis-Menten plot and kinetic parameters, *K_m_, V_max_*, and *k_cat_*, for Grx. *L. monocytogenes* thioredoxin A, TrxA that has efficient thiol-disulfide oxidoreduction activity, was taken as a reference control in this assay. Data are expressed as means ± SDs.

The bioinformatic analysis indicated that *L. monocytogenes* Lmo2344 contains a non-canonical CHYC active motif that slightly differs from the classical CPYC or CGFS active motif of the glutaredoxin homologues in other bacteria species (Figure 1(a)). Phylogenetic analysis further illustrated that Lmo2344 and its homologues from *L. innocua* and *L. grayi* were in the same sub-branch, which are relatively close to the glutaredoxins from *B. subtilis* (Grx and GrxC), *P. multocida* (GrxD), *K. pneumoniae* (GrxD), *S. flexneri* (GrxD), and *P. aeruginosa* (Grx). Nevertheless, the other glutaredoxin homologous from *S. typhimurium* (GrxA), *S. flexneri* (GrxA), *V. cholerae*, and *E. coli* (GrxA) formed a separate clade (Figure 1(b)), suggesting that *L. monocytogenes lmo2344* might encode a putative *B. subtilis*-like glutaredoxin, showing a closer relationship with the CGFS-type glutaredoxin. Using DTT as an electron donor, the recombinant Lmo2344 is able to catalyze the reduction of insulin that contains disulfide bonds (Figure 1(c,d)), however, which was less efficient than the thioredoxin of *L. monocytogenes* (TrxA) under the same conditions (Figure 1(d)). In addition, the mutant proteins Grx_CPYC_ and Grx_CGFS_ exhibited the comparable activity with the wild-type Grx (Figure 1(d)). Therefore, these results demonstrated that Lmo2344 with a non-canonical dithiol-active centre might be a novel glutaredoxin compared to the classical members from other bacteria.

### Deletion of grx did not affect bacterial in vitro growth, but slightly decreased the bacterial colony size

**Figure 2.**
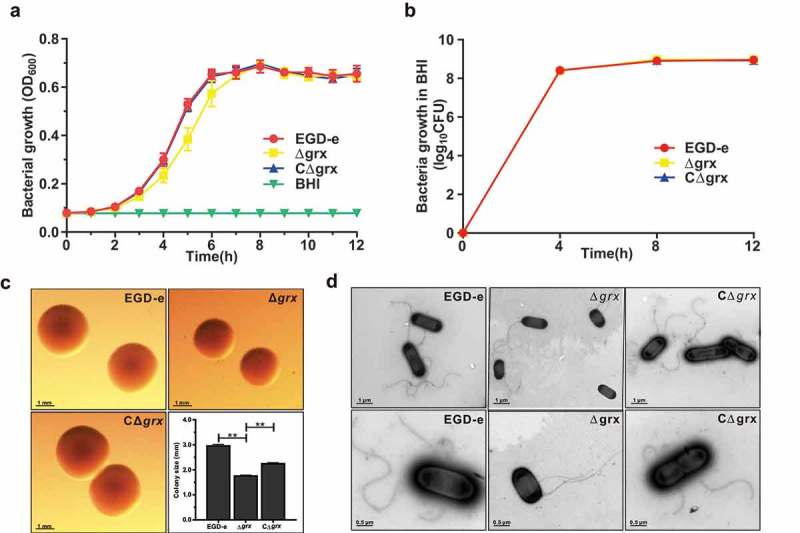
Deletion of *grx* did not affect bacterial *in vitro* growth and motility, but slightly decreased the bacterial colony size. (a-b) *In vitro* growth and colony size assay of *L. monocytogenes* wild-type EGD-e, and gene deletion and complementation mutants (Δ*grx* and CΔ*grx*). Overnight-grown bacteria were washed and diluted (1:100) in fresh BHI broth (a) or BHI agar medium (b), and incubated at 37°C for 12 hours. Kinetic growth at OD_600 nm_ (a) was measured at 1-h intervals, and bacterial CFU numbers (b) counted at 4-h intervals. Data are expressed as means ± SDs. (c) The bacterial colony morphology grown on the BHI agar plates for 12 hours were observed by using a stereo-microscope. The size of the single bacterial colony was measured of 100 bacteria and data are expressed means ± SDs. **P < 0.01. (d) Bacterial flagellar formation observed by transmission electron microscopy (TEM) were performed on soft agar (0.25%) at 30°C.

The Trx and the Grx systems, as the two major antioxidant systems in organisms, play roles in controlling cellular redox environment [2]. Based on our previous research, TrxA is involved in the regulation of oxidative stress and bacterial virulence [8]. Hence, the *grx* gene in-frame deletion mutant strain was developed for revealing the unknown biological functions. As indicated, the *grx* mutant strain exhibited a little lower absorbance at OD of 600 nm compared with the reference strain EGD-e and the complement strain CΔ*grx* during the exponential phase growth in normal BHI, while the counted CFU numbers of the parent and mutant strains are nearly identical (Figure 2(a,b)). These observations showed that lack of this gene had no influence on bacterial growth *in vitro*. However, by using the stereo-microscope observation, the colony size of the *grx* deletion mutant was smaller than the wild-type and complement strains, which was further confirmed by the transmission electron microscopy. This might account for the slight difference in the optical density measured on the bacterial growth, but actually with the same growth ability of these two strains (Figure 2(c,d)). In addition, we also found that absence of *grx* did not affect the flagellar synthesis during the *in vitro* adaption (Figure 2(d)). In conclusion, deletion of *grx* showed no influence on bacterial growth, but slightly decreased the bacterial colony size.

### Deletion of grx remarkably increased tolerance to the oxidizing oxidants, copper, cadmium and diamide, but not to the hydrogen peroxide

**Figure 3.**
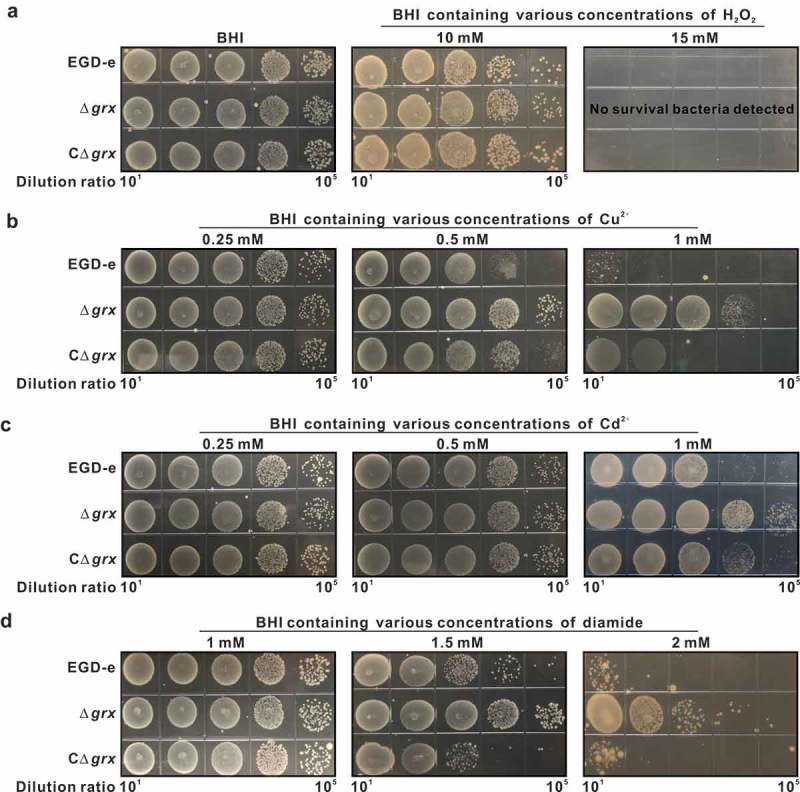
Deletion of *grx* remarkably increased tolerance to the oxidizing oxidants, copper, cadmium and diamide, but not to the hydrogen peroxide. (a-d) Growth of *L. monocytogenes* wild-type EGD-e, and gene deletion and complementation mutants (Δ*grx* and CΔ*grx*) in BHI agar medium supplemented with or without oxidative agents. Overnight-grown bacteria were subjected to hydrogen peroxide (a), copper (b), cadmium (c), and diamide (d) by spotting 10^8^ CFU of each bacterial strain onto BHI plates containing various concentrations of oxidizingoxidants.

Based on the fact that Grx usually exerts antioxidant activity in cells, and further to determine whether *L. monocytogenes* Grx system could contribute to oxidative tolerance, the *L. monocytogenes* EGD-e and the *grx* mutant dstrains (Δ*grx* and CΔ*grx*) were exposed to different oxidative agents. For oxidative stress, H_2_O_2_ was used as a direct oxidant and diamide as a thiol-specific oxidizing agent while the divalent metal ions such as copper and cadmium were used as the redox-active stress. As shown in Figure 3, the parent strain EGD-e and the mutants appeared the similar resistance to hydrogen peroxide under the sub-lethal concentration of 10 mM (Figure 3(a)). When the concentration of H_2_O_2_ increased to 15 mM, no survival bacteria were detected, suggesting that deletion of *grx* significantly enhanced bacterial survival in the presence of the sublethal concentrations of H_2_O_2_ (Figure 3(a)). However, to our surprise, mutation of *grx* rendered this bacterium significantly more resistant to copper, cadmium and diamide, with the increased 2–4 logs in survival bacteria when exposed to the various concentrations of these three oxidative agents (Figure 3(b–d)). Such phenotypes of the deletion mutant can be partially rescued in the complement strain CΔ*grx* (Figure 3(b–d)). In addition, the transcriptomic data revealed that 282 genes or 279 genes show 2-fold higher or lower transcript levels in the Δ*grx* than the parent EGD-e under oxidative conditions (exposure of stationary cells to 4 mM diamide for 1 h) (Table S2 and S3). It is worth noting that some oxidoreductases, like pyruvate oxidase, cytochrome quinol oxidases and glutathione reductase, showed signiﬁcantly higher expression in the Δ*grx* mutant than the wild-type strain (Table 1).

**Table 1.** The transcriptional changes of the oxidative tolerance-related genes in *L. monocytogenes* wild-type EGD-e and Δ*grx* mutant.

Gene name	Annotation	Fold change (Δ*grx*/EGD-e)	Significance
*mo0722*	pyruvate oxidas	9.98	Yes
*qoxA*	cytochromec quinol oxidase (QoxA)	5.74	Yes
*qoxB*	cytochromec quinol oxidase(QoxB)	2.45	Yes
*lmo1433*	glutathione reductase	4.52	Yes

Note: The corresponding model indicates the particular model in our study that corresponds to the model in the existing study. BM, ISBM, DSBM, DISBM are the acronyms of movie attendance models in Section 2.

Taken together, these unexpected findings suggested that *L. monocytogenes* glutaredoxin plays an extraordinary role in bacterial oxidative resistance that counterposes the classical fact that the presence of the Grx system in *E. coli* provides a strong backup for the Trx system to participate in the antioxidant process [20]. This is also the first report to determine that absence of glutaredoxin instead increases the oxidant tolerance in bacterial species *via* upregulating some oxidative tolerance-related oxidases.

### Mutation of grx increased the efficiency of bacterial intracellular invasion and growth but not cell-to-cell spreading via upregulating the internalins, InlA and InlB

**Figure 4.**
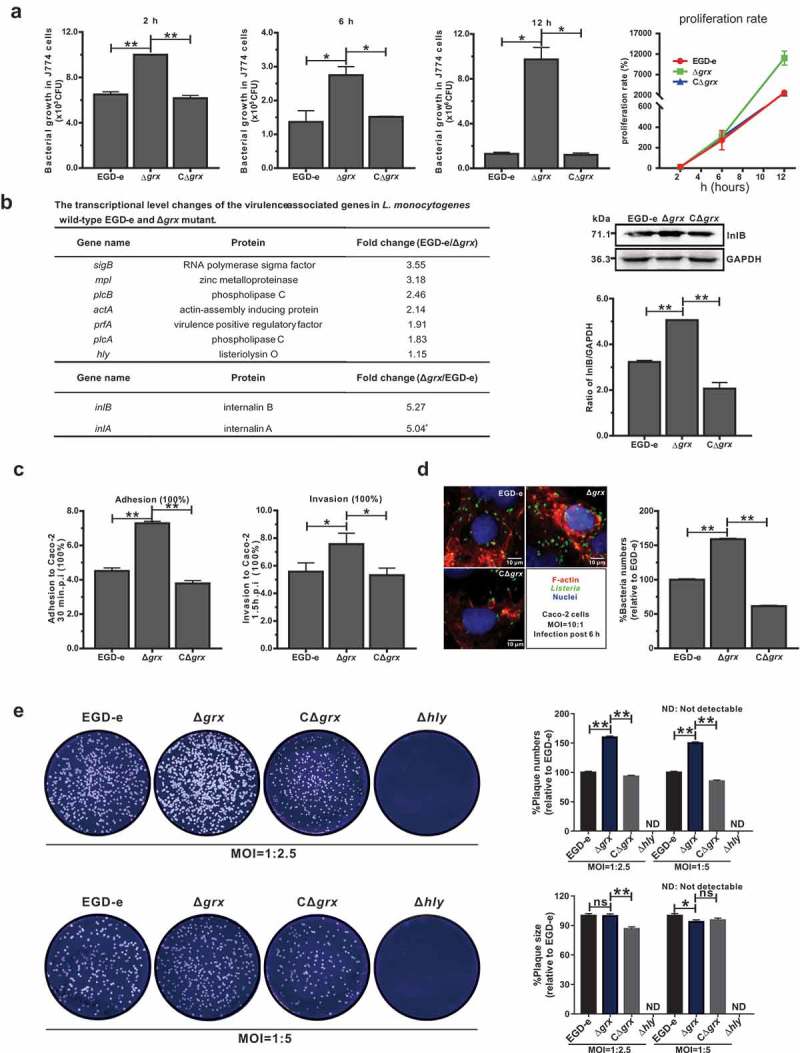
Mutation of *grx* increased the effciency of bacterial intracellular growth and cell-to-cell spreading *via* upregulating the internalins, InlA and InlB. (a) Intracellular growth of *L. monocytogenes* in murine-derived J774 macrophages. Gentamycin (50 μg/mL) was added 30 min post infection. The J774 cells infected with *L. monocytogenes* wild-type EGD-e and *grx* mutant were lysed at the indicated time points (2, 6, and 12 h), and viable bacteria serially plated on BHI plates. The number of recovered bacteria able to invade cells and survive are expressed as means ± SDs for each strain. (b) The transcriptional level changes of the virulence-associated factors (*prfA, plcA, plcB, hly, mpl, inlA* and *inlB*) of *L. monocytogenes* wild-type EGD-e and *grx* mutant, which were identified by the transcript analysis. Expression of InlB in the wild-type and mutant strains were assayed by Western blotting. (c) Adhesion and invasion of *L. monocytogenes* in human epithelial cells, Caco-2. Cells infected with *L. monocytogenes* wild-type EGD-e, and gene deletion and complementation mutants (Δ*grx* and CΔ*grx*) at the indicated time points were lysed and viable bacteria serially plated on BHI plates. The number of recovered bacteria able to invade cells and survive are expressed as means ± SDs for each strain. (d) Intracellular multiplication of *L. monocytogenes* in Caco-2 cells 6 h post infection. Bacteria were detected with anti-Lm (green), and bacteria actin tails and host actin were detected using phalloidin (red), while the cell nucleus was labeled with DAPI (blue). The scale bar is 10 μm. The high-magnification images displayed at the bottom of each image show F-actin (red), bacteria (green), and nuclei (blue). (e) Plaque assay performed on the L929 fibroblast monolayers infected by *L. monocytogenes* wild-type EGD-e, and gene deletion and complementation mutants (Δ*grx* and CΔ*grx*). The plaque numbers of the mutant strains were indicated as a percentage of those formed by the wild-type strain. The mutant strain *hly*, which is completely unable to spread during cell infection, was taken as a reference negative control. *P<0.05; **P<0.01; ns means no significance. Data are expressed as means ± SDs.

In view of the findings on the unusual roles of *L. monocytogenes* Grx during the adaption under oxidative stress as illustrated above, we attempted to further explore the functions that performed by Grx during the bacterial intracellular infection. The murine-derived macrophages J774 cells were employed to mimic the host oxidative environments. Unexpectedly, the *grx* mutant showed more efficient to proliferate inside the macrophages 2, 6 and 12 hours post infection compared with the parent strain EGD-e and the complement strain CΔ*grx* (Figure 4(a)), which was consistent with our previous findings on the increased resistance of *grx* mutation under the oxidative stress. Moreover, we compared the transcriptional changes of the *Listeria* virulence genes (*prfA, hly, plcA, plcB, mpl, inlA*, and *inlB*) between the wild-type and Δ*grx* deficient strains. The data revealed most of the virulence genes did not exhibit significant alteration in the transcript levels, with exception of the two internalins (*inlA* and *inlB*) whose transcripts were approximately upregulated 5-fold in the Δ*grx* mutant (Figure 4(b)). In addition, the blotting data indicated that expression of InlB was markedly increased in the absence of Grx (Figure 4(b)), which further validated the regulatory roles of Grx on expression of *Listeria* interalin. However, due to the failure to obtain the specific poly-antibodies against InlA we were unable to conduct the similar experiments on expression changes of InlA in the *grx-*deleted mutant. As the fact indicates that *L. monocytogenes* two surface proteins, InlA and InlB are employed by the bacteria to invade mammalian cells *via* cadherins transmembrane proteins and Met receptors respectively [21]. To determine the roles of Grx in bacterial invasion, the epithelial cells Caco-2 were used to compare the wild-type and *grx* mutant strains in terms of adhesion, invasion, and intracellular survival. The *grx* mutant was significantly more adhesive and invasive, compared to that of the wild-type and complement strains (Figure 4(c)). This was further confirmed in an intracellular multiplication assay where the *grx* deletion mutant with more intracellular bacteria numbers were more efficient to escape from the vacuolar and multiply intracellularly in Caco-2 cells compared to that of the wild-type EGD-e and the complemented strain (Figure 4(d)). To further assess whether Grx played a part in cell-to-cell spreading efficiency, we tested the ability of *grx* mutant and wild-type strain EGD-e to form plaques on L929 fibroblast monolayers after 54 hours of infection in the presence of a low concentration of gentamycin. As indicated in Figure 4, the number of plaques produced by Δ*grx* was significantly higher than its parent and complement strains while the plaque sizes produced by Δ*grx* were slightly decreased compared to the wild-type (Figure 4(e)), suggesting that the ability of this bacterium to spread among cells was not affected by the deletion of this gene. Collectively, these data suggested Grx was not required for bacterial infection inside the host cells, and instead, deficient of this gene can remarkably increase the invasion and intracellular survival of this bacterium, through upregulating the transcript levels of internalin A and B.

### Deletion of grx enhanced the bacterial virulence in mice

**Figure 5.**
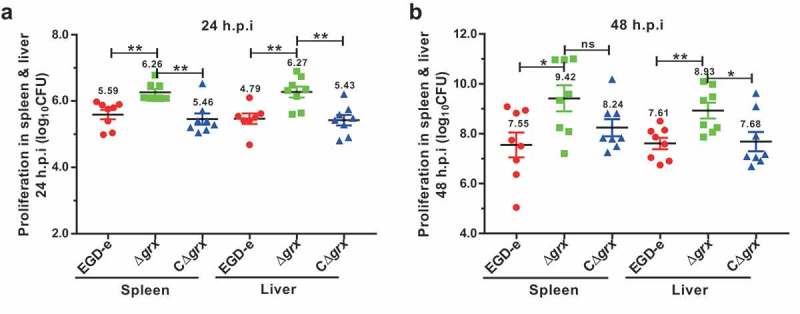
Deletion of *grx* resulted in the increased virulence in mice. Proliferation of *L. monocytogenes* in mice organs. The *L. monocytogenes* wild-type and mutant Δ*grx* bacteria were inoculated intraperitoneally into ICR mice at ~4 × 10^6^ CFU. Animals were euthanized 24 (a) and 48 (b) hours post infection, and organs (livers and spleens) were recovered and homogenized. Homogenates were serially diluted and plated on BHI agar. Numbers of bacteria colonized in liver and spleen are expressed as means ± SDs of the log_10_CFU per organ for each group. *P<0.05; **P<0.01; ns means no significance.

Having observed an intensified intra-macrophage growth in the Δ*grx*-suffered macrophages prompted us to further explore the virulence of this mutant strain in mice. The ICR mice were challenged intraperitoneally with ~4 × 10^6^ CFU of the wild-type and Δ*grx* mutant strains. As indicated, the number of colony-forming units (CFU) recovered from the spleens and livers of infected mice at 24 (about 2-fold from spleens and livers) and 48 (about 10-fold from spleens and livers) hours of infection was significantly higher for the Δ*grx* mutant compared with the wild-type strain (Figure 5). Such enhanced bacterial pathogenicity in the *grx* mutant can be fully restored in the complement strain, demonstrating that the Δ*grx* mutant strain exhibited a reinforced virulence in mice. Combined with the results from the cell experiments, these data consistently determined that the pathogenicity of Δ*grx* mutant was enhanced, and that mice infected with this mutant exhibited higher bacterial burdens compared with the mice infected with the wild-type strain. Taken together, these results established a surprising role of glutaredoxin in the virulence of *L. monocytogenes*.

## Discussion

Glutaredoxin system, together with the thioredoxin system are the major cellular disulfide reductases in cells, existing in most of living organisms from prokaryotes, plants, virus, and eukaryotes [22,23]. Glutaredoxin can efﬁciently reduce glutathione mixed disulﬁdes that have been formed by nucleophilic attack of GSH on disulﬁde bonds. In such a reaction, glutaredoxins reduce the protein-GSH mixed disulﬁde, in the process forming a Grx-GSH intermediate that is subsequently reduced by a second molecule of glutathione [24]. Moreover, under oxidative stress, glutaredoxin may catalyze formation of mixed disulfides from GSSG, which may be of a protective function to avoid oxidation of a thiol to higher oxidation state [25]. Unlike with the fact that the bacterial thioredoxins have been widely shown to play major roles in protection of cells against toxic oxygen species as well as maintaining the intracellular thio-disulﬁde balance [8,26], limited data had been available on the role of the glutaredoxins. However, glutaredoxins from *E. coli* has previously been demonstrated to contribute to the defense against oxidative stress. *E. coli* mutants that lack Grx3, but not those lack Grx1 or Grx2, are becoming more sensitive to hydrogen peroxide and thus killed more efficiently by oxidizing agents [14], which therefore prompted us to investigate the roles of glutaredoxin in *L. monocytogenes*, a stress well-adapted foodborne pathogen. In our study, we suggest an extraordinary role of *L. monocytogenes* glutaredoxin involved in the defense against oxidative stress and bacterial virulence during host infection, which is the first time to demonstrate the biological functions of Grx in gram-positive bacteria.

**Figure 6.**
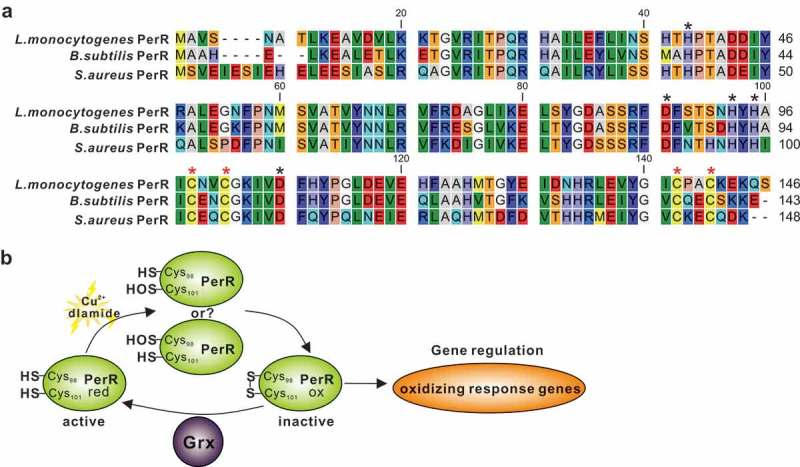
A proposed model for *L. monocytogenes* Grx as a novel glutaredoxin in bacterial oxidative tolerance. (a) Based on sequence alignment using the full-length sequences of PerRs from *B. subtilis* and *S. aureus* as templates, four cysteine residues (C98, C101, C138 and C141) that forms a high-affinity Zn^2+^ binding site (Cys_4_Zn), and five other residues (H39, D87, H93, H95 and D106) that preferentially binds Fe^2+^ or Mn^2+^ are found to be perfectly conserved in *L. monocytogenes* PerR. These residues are indicated with asterisks. (b) Therefore, we here proposed that *L. monocytogenes* repressor PerR could be irreversibly inactivated when bacteria exposed to oxidative conditions and failed to switch back to the activated form in the absence of Grx, resulting in derepression of the PerR-regulated oxidizing response genes.

As previously reviewed, thioredoxin is ubiquitous in bacteria, whereas the Grx antioxidant system is lacking in some specific bacteria [22]. *E. coli* has been extensively studied and is a modern bacterium with Trx and Grx systems. The presence of the Grx system in *E. coli* provides a strong backup for the Trx system to participate in the antioxidant process by deglutathionylation as in mammalian cells, which was nevertheless not the case in our finding on *L. monocytogenes*. Our previous work has collectively determined that *L. monocytogenes* thioredoxin A as a vital cellular reductase is essential for maintaining a highly reducing environment in the bacterial cytosol, which provides a favorable condition for protein correct-folding, and therefore contributes to bacterial antioxidant and virulence [8]. On the contrary, we here in this study found that the absence of Grx remarkably rendered this pathogen more tolerant to the oxidizing environment, which suggested that this enzyme might be redundant during the adaption in the oxidative conditions. Back to the sophisticated model of *E. coli*, glutaredoxin participates in the process of antioxidant mainly *via* redox modification on the oxidative stress activated transcription factor OxyR, which acts as a peroxide-sensing regulator and positively controls expression of many antioxidant proteins, such as KatG, TrxB, AphC and SoxR [3,27,28]. Under oxidative stress, the cysteine residues in OxyR (Cys199 and Cys208) can be oxidized or *S*-glutathionylated to form an intramolecular disulfide bond, resulting in conformational changes that enable OxyR to function as a transcriptional activator and initiate transcription of the following regulated antioxidant genes. Glutaredoxin 1 subsequently deactivates OxyR by reducing the disulfide bond or de-glutathionylation, forming an autoregulating response process [3,29]. However, the Gram-positive bacteria often employ a different oxidative stress regulator PerR, with *L. monocytogenes* no exception. The PerR, a family member of transcriptional repressor and best characterized in *B. subtilis*, can be inactivated by oxidation to trigger transcription of the oxidative response genes [30,31]. In *S. aureus* the *perR* mutant demonstrates increased resistance to hydrogen peroxide, most likely through increased expression of catalase [32]. Similarly, a previous study has firstly determined that eliminating *L. monocytogenes* PerR resulted in a mutant which was more resistant to the action of H_2_O_2_ and exhibited higher transcription of the PerR-regulated genes, including *fur, trxB, kat*, and *hemA* [33,34]. Based on sequence alignment using the full-length sequences of PerRs from *B. subtilis* and *S. aureus* as templates [30,31], four cysteine residues (C98, C101, C138 and C141) that forms a high-affinity Zn^2+^ binding site (Cys_4_Zn), and five other residues (H39, D87, H93, H95 and D106) that preferentially binds Fe^2+^ or Mn^2+^ are found to be perfectly conserved in *L. monocytogenes* PerR (Figure 6(a)). Therefore, we here proposed that *L. monocytogenes* repressor PerR could be irreversibly inactivated when bacteria exposed to oxidative conditions and failed to switch back to the activated form in the absence of Grx, resulting in derepression of the PerR-regulated oxidizing response genes (Figure 6(b). This might be a comparatively reasonable explanation for the fact that deletion of *grx* generated a remarkably increased response of *L. monocytogenes* to oxidative stress. More important, in this study, the cytochrome quinol oxidase A, and B (QoxA and QoxB), showed signiﬁcantly higher expression in the Δ*grx* mutant strain than the wild-type. Relevant to patho-physiology, cytochrome oxidases are expressed in a number of bacterial pathogens. These include *Salmonella* [35], *Mycobacterium tuberculosis* [36], *Shigella flexneri* [37], *Streptococcus* [38], and *L. monocytogenes* [39,40]. The cytochrome oxidases have been studied to play a role of in bacterial protection against oxidative stress. In *E. coli*, mutants defective in cytochrome oxidase display increased levels of intracellular H_2_O_2_, and show an increased sensitivity to external H_2_O_2_ [41]. Particularly interesting is also the case of *Brucella abortus*, in which the lack of cytochrome oxidases expression is associated not only with an increased sensitivity to oxidative stress [42], but also with a reduced bacterial virulence in a murine infection model. Overall, the findings suggest that cytochrome oxidases from several bacterial species are able to afford protection against oxidative stress, and this may represent an advantage particularly for pathogenic bacteria that have to cope with the hostile oxidative conditions during host infections. These data imply that upregulation of expression of these oxidases in the mutant strain Δ*grx* of *L. monocytogenes* might contribute signiﬁcantly to the enhanced oxidative resistance ability.

More importantly, we found that lack of *grx* rendered *L. monocytogenes* more efficient to invade during infection on host cells and mice model by upregulating InlA and InlB. Generally, bacteria express a range of disulfide-bonded virulence factors, including secreted toxins and surface components. To ensure functional activity, many of these proteins must be oxidatively folded. The thioredoxin super-family system is the major cellular disulﬁde reductase in cells, which can provide a highly reducing environment and then function as an eﬀector to facilitate correct folding for proteins containing disulfide bond(s). Among the oxidoreductase members, the Trx system is necessary for the virulence of many bacterial pathogens, including *H. pylori, M. tuberculosis, F. novicida, N. gonorrhoeae* and *B. fragilis* [26,43–46]. Likewise, we previously demonstrated *L. monocytogenes* employs a classical thioredoxin, TrxA that contributes to bacterial pathogenicity mainly through participating in the correct folding and activation of the virulence regulator PrfA whose activation is highly dependent on the reducing environment [8,47,48]. No expression or constitutive overexpression of TrxA *in vivo* would disturb the equilibrium of the intracellular redox potential and thus bring harm to *L. monocytogenes* for its ability to establish infections [8]. However, much less is known about the role of Grxs involved in bacterial *in vivo* infection. Our novel findings on *L. monocytogenes* glutaredoxin suggest this oxidoreductase might have functional redundancy in bacterial infection, and instead, depletion of *grx* resulted in higher efficiency of this pathogen to infect host cells. Nevertheless, we further observed that the absence of *grx* did not affect the transcription and expression profiles of the main *Listeria* virulence-associated factors, including PrfA, Listeriolysin O, phospholipases, ActA, with exception of the two internalins (*inlA* and *inlB*) whose transcripts were approximately upregulated 5-fold in the Δ*grx* mutant. As the known fact tells that expression of inlA and inlB is regulated by both PrfA-dependent and -independent mechanisms. In addition, expression of InlA was not strictly dependent on the presence of the PrfA regulator protein. Transcription analysis of the *inlAB* locus revealed that the *inlA* gene was transcribed by several promoters, of which only one is PrfA dependent [49]. In the present study, we indicated that *prfA* was transcriptionally downregulated (1.91-fold change, no significance) in the absence of Grx while *inlA* and *inlB* were significantly upregulated, suggesting that regulation of InlA/InlB by Grx might be in a PrfA-independent manner. Moreover, the global regulator SigB has been determined to contribute to positively controlling expression of *inlA* and *inlB* [50]. Combined with the findings showing that *sigB* was transcriptionally downregulated (3.55-fold change) in the absence of Grx, we therefore speculated that regulation of InlA/InlB by Grx might be SigB-independent. Collectively, we here suggested a hypothesis that Grx contributes to negative regulation of these two internalins *via* Grx in a direct manner or *via* an unknown regulator in an indirect manner. In *L. monocytogenes*, PrfA is exclusively activated in the cytosol of host cells, leading to the assumption that the activating cofactor for PrfA is specific to this compartment [47]. Recently, glutathione (GSH), either generated by bacteria or derived from host cells, was found to be the essential small molecule cofactor of PrfA through allosteric binding to the protein [48]. Therefore, Grx, as a reducing enzyme, might contribute to provide a highly reducing environment for reduced PrfA maintaining, and therefore acts as an activating co-factor, thereby providing a reasonable explanation for our finding that deletion of *grx* resulted in a relatively slight downregulation of several PrfA-regulated virulence genes (*mpl, plcB* and *actA*), which might also be a better attribution for the slightly-decreased plaque size produced by the *grx* mutant bacteria.

In general, stress-tolerant *Listeria* strains are more invasive *in vitro* [51] and more virulent *in vivo* [52]. Obviously, the capacity to tolerate oxidative stress is related to the virulence potential of *L. monocytogenes* and other bacterial species [53]. Consequently, we speculate that the unexpected alteration in pathogenicity of *grx* mutant could be attributed to the increased resistance in oxidative stress. In summary, we here for the first time demonstrated that *L. monocytogenes* glutaredoxin plays a counterintuitive role in bacterial oxidative resistance and intracellular infection, which is the first report to provide valuable evidence for the role of glutaredoxins in bacterial infection, and more importantly suggests a favorable model to illustrate the functional diversity of glutaredoxin systems in bacteria during environmental adaption and host infection. More importantly, the future study should be needed for better understanding the molecular insights of glutaredoxin system employed by *L. monocytogenes* to adapt to niche environments outside and inside the host.
