# Supplementary material for: Deletion of glutaredoxin promotes oxidative tolerance and intracellular infection in Listeria monocytogenes
Source: Virulence. 2019 Nov 2;10(1):910–24. doi: 10.1080/21505594.2019.1685640 (PMC6844310; doi:10.1080/21505594.2019.1685640)
Supplement: Supplemental Material [file kvir-10-01-1685640-s001.zip › Grx supporting Figure S1.pdf]

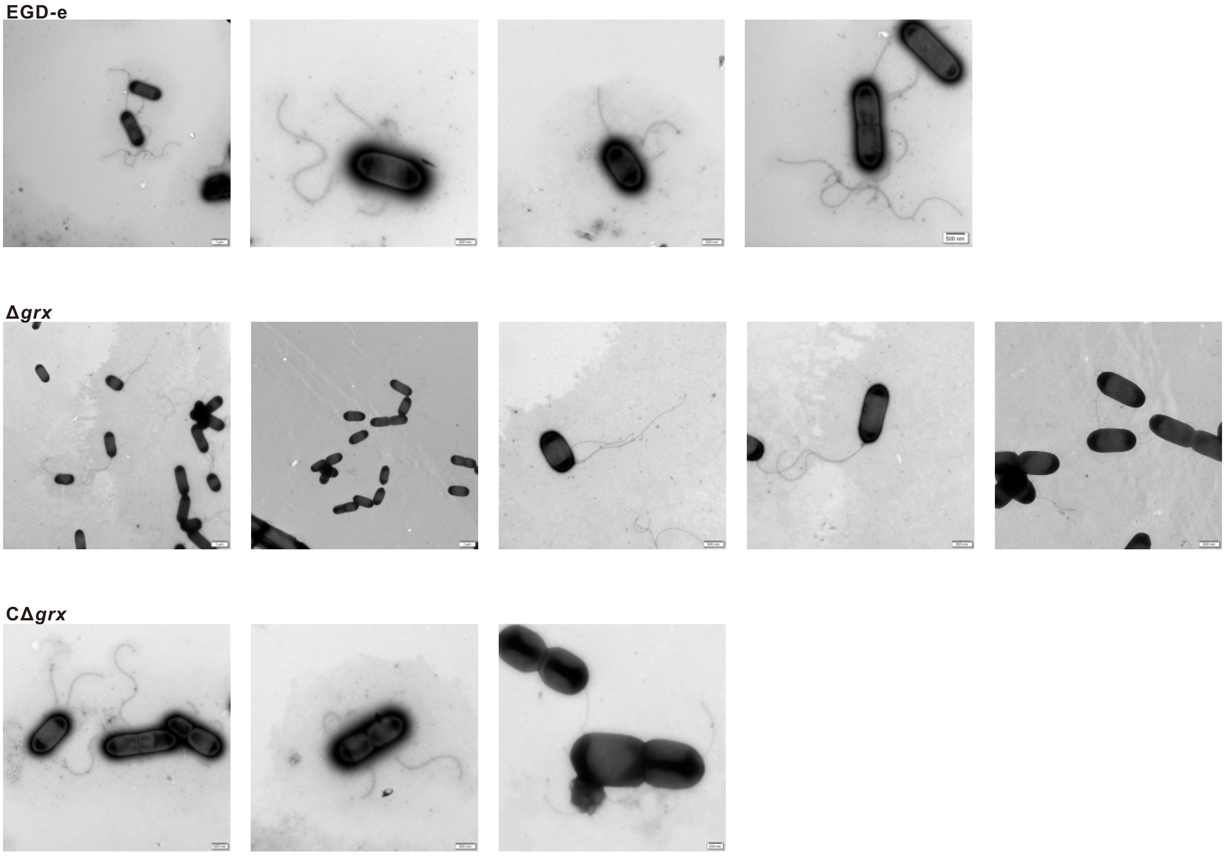

Figure S1. All the original images of Figure 2D observed by transmission electron microscopy (TEM).
