# Supplementary material for: Deletion of glutaredoxin promotes oxidative tolerance and intracellular infection in Listeria monocytogenes
Source: Virulence. 2019 Nov 2;10(1):910–24. doi: 10.1080/21505594.2019.1685640 (PMC6844310; doi:10.1080/21505594.2019.1685640)
Supplement: Supplemental Material [file kvir-10-01-1685640-s001.zip › Grx supporting Table S1.pdf]

Table S1. The PCR primers used in this study. The nucleotides introduced to create restriction enzyme sites are underlined. All primers were synthesized by GENEWIZ Inc., Suzhou, China.

Primer names	Primer sequences (5'-3')	Products (bp)	Descriptions
Δ*grx*-a	CCGGAATTCGAAGTGCATCGAGCGCGAAAC	528	Used for construction of *grx* null mutant
Δ*grx*-b	TACTTCCCCCTTTTCAACGACGACATTAGCCATGGACAACT		
Δ*grx*-c	GCTAATGTCGTCGTTGAAAAGGGGGAAGTAAAATGACTAGAAAAGAA	496	
Δ*grx*-d	TGGCTGCAGGCAATAGTGTAACGATCGCTGTGGG		
Grx-exp-fwd	GGAATTCCATATGGCTAATGTCGTCGTTTGGAGTAAAG	283	Used for overexpression of the recombinant Grx in *E. coli*
Grx-exp-rev	CCGCTCGAGTTTTACTTCCCCCTTTTCAAACAATGC		
Grx_H13P-fwd	GTTTGGAGTAAAGAAGGGTGTCCTTATTGTAAAGATGTCAAAGATTTT	5502	Used for the site-directed mutagenesis of Grxs (Grx_{CPYC} and Grx_{CGFS})
Grx_H13P-rev	AAAATCTTTGACATCTTTACAATAAGGACACCCTTCTTTACTCCAAAC		
Grx_H13GY14FC15S-fwd	GTTTGGAGTAAAGAAGGGTGTGGTTTTTGTAAAGATGTCAAAGATTTT		
Grx_H13GY14FC15S-rev	AAAATCTTTGACATCTTTACAAAAACCACACCCTTCTTTACTCCAAAC		
CΔ*grx*-a	TCCGAGCTCGTTGAAAACTTCCCGTATTAACCACCGTATA	324	Used for complementation of the *grx* deletion mutant under its native promoter
CΔ*grx*-b	GACGACATTAGCCATCAAATCGCCCCTTTTCAATCCCTAT		
CΔ*grx*-c	AAAAGGGGCGATTTGATGGCTAATGTCGTCGTTTGGAGTAAAGA	294	
CΔgrx-d	TCCCCCGGGTCATTTTACTTCCCCCTTTTCAAACAATG		
16SrRNA-RT-fwd	TGAAATGCGTAGATATGTGGAGG	129	Used for RT-PCR
16SrRNA-RT-rev	ATCGTTTACGGCGTGGACTA		
prfA-RT-fwd	TGCGGTCAACTTTTAATCCTG	112	
prfA-RT-rev	CGATGCCACTTGAATATCCTAACT		
mpl-RT-fwd	GGAAGTTGCGAGTTGGATTGT	98	
mpl-RT-rev	TGTGGGTATCAGCCCGTTC		

plcA-RT-fwd	AGCCATTAGTCATTAACTCACGC	88
plcA-RT-rev	ACACTCGGACCATTGTAGTCATC	
plcB-RT-fwd	AAAGGTGGTTCTAGGTATGTGCTT	101
plcB-RT-rev	GGAGCTGCGGGTGTTTGTA	
hly-RT-fwd	ACTGGTTTAGCTTGGGAATGG	107
hly-RT-rev	TATTTCGGATAAAGCGTGGTG	
inlA-RT-fwd	GGTCTCACAAACAGATCTAGACCAAGT	122
inlA-RT-rev	GTTGATTATTGCTGAAATTTATTTGTG	
inlB-RT-fwd	GAGACTATCACCGTGCCAACG	112
inlB-RT-rev	TTTGTGTCACTGCATCTGTCACAC	
