# Supplementary material for: Deletion of glutaredoxin promotes oxidative tolerance and intracellular infection in Listeria monocytogenes
Source: Virulence. 2019 Nov 2;10(1):910–24. doi: 10.1080/21505594.2019.1685640 (PMC6844310; doi:10.1080/21505594.2019.1685640)
Supplement: Supplemental Material [file kvir-10-01-1685640-s001.zip › Grx supporting Table S2.pdf]

Table S2. Genes identified by transcriptome analysis as significantly up-regulated in *L. monocytogenes* Δ*grx* mutant.

Gene name	Annotation	Fold change (Δ*grx*/EGD-e)	Significance
lmo2255	hypothetical protein	∞	Yes
lmo0351	mannnose-specific phosphotransferase	∞	Yes
lmo0418	hypothetical protein	∞	Yes
lmo1180	ethanolamine utilization protein	∞	Yes
lmo0350	hypothetical protein	158.03	Yes
lmo0348	dihydroxyacetone kinase	48.67	Yes
lmo0347	dihydroxyacetone kinase	29.97	Yes
opuCD	glycine betaine/L-proline ABC transporter	25.52	Yes
lmo0514	cell wall surface anchor family protein	24.80	Yes
glnR	HTH-type transcriptional regulator	22.75	Yes
lmo0345	ribose 5-phosphate isomerase B	20.50	Yes
opuCC	glycine betaine/L-proline ABC transporter substrate-binding protein	18.67	Yes
lmo0346	triosephosphate isomerase 2	16.99	Yes
opuCB	glycine betaine/L-proline ABC transporter	15.60	Yes
lmo0294	transcription activator of glutamate synthase	14.61	Yes
lmo0723	methyl-accepting chemotaxis protein	13.80	Yes
lmo0586	hypothetical protein	13.32	Yes
lmo1734	glutamate synthase subunit alpha	13.05	Yes
lmo0724	uncharacterized protein yvpB	13.00	Yes
ilvD	dihydroxy-acid dehydratase	12.75	Yes
sepA	metallo-beta-lactamase	12.58	Yes
lmo1243	hypothetical protein	12.22	Yes
lmo1539	glycerol uptake facilitator protein	10.63	Yes
lmo2783	PTS system cellobiose-specific IIC component	10.60	Yes
lmo0977	esterase	10.52	Yes
lmo0722	pyruvate oxidase	9.98	Yes
lmo1516	ammonium transporter	9.96	Yes
lmo1241	hypothetical protein	9.76	Yes
lmo0585	hypothetical protein	9.56	Yes
lmo0591	hypothetical protein	8.32	Yes
lmo0781	PTS system mannose-specific transporter subunit IID	7.98	Yes
lmo0134	acetyltransferase	7.91	Yes
lmo1421	osmoprotectant transport system ATP-binding protein	7.49	Yes
opuCA	glycine betaine/L-proline ABC transporter	7.15	Yes
lmo1517	nitrogen regulatory protein P-II	6.76	Yes

lmo2179	peptidoglycan binding protein	6.39	Yes
lmo0587	secreted hypothetical protein	6.35	Yes
eutB	ethanolamine ammonia-lyase large subunit	6.28	Yes
lmo0944	uncharacterized protein YneR	6.23	Yes
ilvB	acetolactate synthase I/II/III large subunit	6.08	Yes
lmo1248	7,8-dihydro-8-oxoguanine triphosphatase	5.99	Yes
lmo0976	acetyltransferase	5.98	Yes
pduQ	iron-containing alcohol dehydrogenase	5.93	Yes
lmo0343	transaldolase	5.92	Yes
qoxA	cytochrome c quinol oxidase	5.74	Yes
lmo2714	pepdidoglycan bound protein	5.69	Yes
lmo0600	hypothetical protein	5.68	Yes
lmo0301	PTS system beta-glucoside-specific transporter subunit IIA	5.62	Yes
lmo0344	2-deoxy-D-gluconate 3-dehydrogenase	5.56	Yes
lmo0399	PTS system fructose-specific transporter subunit IIB	5.54	Yes
lmo1422	osmoprotectant transport system substrate-binding protein	5.49	Yes
lmo2736	glycerate kinase	5.35	Yes
eutC	ethanolamine ammonia-lyase small subunit	5.33	Yes
lmo0986	antibiotic transport system ATP-binding protein	5.33	Yes
lmo1424	manganese transport protein MntH	5.30	Yes
lmo0398	PTS system fructose-specific IIA component	5.29	Yes
lmo0782	PTS system mannose-specific IIC component	5.29	Yes
inlB	internalin B	5.27	Yes
lmo2437	glyoxalase/bleomycin resistance protein/dioxygenase	5.27	Yes
lmo0602	acetyltransferase	5.22	Yes
inlA	internalin A	5.04	Yes
lmo2749	glutamine amidotransferase	5.01	Yes
glpD	glycerol-3-phosphate dehydrogenase	4.94	Yes
lmo2571	nicotinamidase	4.88	Yes
lmo2085	peptidoglycan binding protein	4.88	Yes
lmo2724	DNA binding 3-demethylubiquinone-9 3-methyltransferase domain-containing protein	4.78	Yes
lmo2463	putative drug exporter of the RND superfamily	4.74	Yes
lmo2570	hypothetical protein	4.70	Yes
lmo0105	chitinase B	4.69	Yes
lmo2735	sucrose phosphorylase	4.61	Yes
lmo0596	hypothetical protein	4.57	Yes
lmo0987	ABC-2 type transport system permease protein	4.54	Yes
lmo2434	glutamate decarboxylase	4.53	Yes

lmo1433	glutathione reductase	4.52	Yes
lmo2697	PTS system mannnose-specific IIA component	4.45	Yes
lmo0321	ATP-dependent DNA helicase RuvA	4.40	Yes
lmo1113	hypothetical protein	4.28	Yes
lmo0358	PTS system fructose-specific IIB component	4.28	Yes
lmo1945	riboflavin transporter FmnP	4.27	Yes
lmo2277	purine nucleoside phosphorylase DeoD-type	4.23	Yes
lmo0880	lysM domain-containing protein	4.22	Yes
lmo2772	PTS system beta-glucosides-specific IIA component	4.13	Yes
lmo2771	6-phospho-beta-glucosidase	4.13	Yes
lmo0573	guanine/hypoxanthine permease pbuG	4.11	Yes
lmo1973	PTS system ascorbate-specific IIA component	4.08	Yes
PdhD	dihydrolipoamide dehydrogenase	4.08	Yes
glpK	glycerol kinase	4.07	Yes
lmo0590	hypothetical protein	4.07	Yes
lmo2696	dihydroxyacetone kinase subunit DhaL	4.03	Yes
lmo0610	hypothetical protein	4.02	Yes
lmo0439	hypothetical protein	4.00	Yes
lmo2178	peptidoglycan binding protein	3.99	Yes
lmo2603	amidase	3.92	Yes
lmo1181	ethanolamine utilization cobalamin adenosyltransferase	3.91	Yes
hisZ	ATP phosphoribosyltransferase regulatory subunit	3.90	Yes
lmo2099	mannitol operon transcriptional antiterminator	3.89	Yes
lmo0588	deoxyribodipyrimidine photolyase	3.88	Yes
lmo1518	hypothetical protein	3.86	Yes
lmo2572	dihydrofolate reductase	3.85	Yes
lmo0027	PTS system beta-glucoside-specific transporter subunit IIABC	3.84	Yes
lmo1150	transcriptional regulator PocR	3.83	Yes
lmo0601	hypothetical protein	3.81	Yes
serC	phosphoserine aminotransferase	3.81	Yes
lmo0019	N-acetylmuramoyl-L-alanine amidase	3.74	Yes
lmo2773	beta-glucoside operon transcriptional antiterminator	3.70	Yes
ulaA	PTS system ascorbate-specific transporter subunit IIC	3.70	Yes
lmo2734	glycosyl hydrolase	3.66	Yes
lmo0160	peptidoglycan binding protein	3.65	Yes
lmo1568	integral membrane protein	3.65	Yes
lmo0794	hypothetical protein	3.61	Yes
lmo2233	LysR family transcriptional regulator	3.61	Yes
lmo1432	hypothetical protein	3.60	Yes

lmo2210	hypothetical protein	3.59	Yes
lmo1974	GntR family transcriptional regulator	3.56	Yes
lmo1885	Xanthine phosphoribosyltransferase	3.54	Yes
lmo2098	PTS system galactitol-specific enzyme IIA component	3.49	Yes
lmo0265	succinyl-diaminopimelate desuccinylase	3.47	Yes
lmo1187	ethanolamine utilization protein EutQ	3.46	Yes
gltC	transcription activator of glutamate synthase operon GltC	3.45	Yes
lmo2436	beta-glucoside operon transcriptional antiterminator	3.42	Yes
lmo0989	transcriptional regulator for hemolysin	3.40	Yes
lmo2346	polar amino acid transport system ATP-binding protein	3.34	Yes
lmo2573	alcohol dehydrogenase	3.32	Yes
lmo2713	GW repeat-containing protein	3.30	Yes
lmo0642	putative membrane protein	3.30	Yes
lmo0995	acyltransferase	3.28	Yes
inlH	internalin H	3.27	Yes
lmo1246	ATP-dependent RNA helicase DbpA	3.23	Yes
lmo2397	NifU family protein	3.22	Yes
lmo2708	PTS system cellobiose-specific IIC component	3.22	Yes
lmo0783	PTS system mannose-specific EIIAB component	3.17	Yes
lmo1864	hemolysin III	3.16	Yes
lmo0555	proton-dependent oligopeptide transporter	3.10	Yes
lmo0784	PTS system mannose-specific IIA component	3.10	Yes
ilvH	acetolactate synthase 3 regulatory subunit	3.08	Yes
pdhC	dihydrolipoamide acetyltransferase	3.07	Yes
lmo0991	tellurite resistance protein TerC	3.06	Yes
lmo1847	manganese/iron transport system substrate-binding protein	3.05	Yes
lmo0669	glucose 1-dehydrogenase	3.01	Yes
lmo1261	hypothetical protein	3.01	Yes
lmo1848	metal cations ABC transporter (permease protein)	3.00	Yes
lmo1849	manganese transport system ATP-binding protein mntB	3.00	Yes
lmo1179	aldehyde dehydrogenase	2.98	Yes
lmo2345	bacterial luciferase	2.97	Yes
hisG	ATP phosphoribosyltransferase catalytic subunit	2.97	Yes
fbp	fructose-1,6-bisphosphatase	2.96	Yes
lmo0107	ABC transporter	2.95	Yes
lmo2396	cell wall surface anchor family protein	2.92	Yes
lmo0554	alcohol dehydrogenase	2.92	Yes

lmo0559	CorA family magnesium transporter	2.92	Yes
lmo2428	Rod shape-determining protein RodA	2.91	Yes
lmo2602	MgtC family protein	2.90	Yes
lmo0654	conserved hypothetical protein	2.90	Yes
lmo1689	hypothetical protein	2.89	Yes
lmo0192	pur operon repressor	2.89	Yes
lmo0300	6-phospho-beta-galactosidase	2.88	Yes
lmo0517	phosphoglycerate mutase family protein	2.87	Yes
lmo0798	Lysine-specific permease	2.83	Yes
lmo0108	ABC transporter	2.83	Yes
lmo0092	ATP synthase subunit beta	2.82	Yes
lmo0576	zinc metalloprotease	2.81	Yes
lmo2462	renal dipeptidase	2.81	Yes
lmo1760	geranylglyceryl phosphate synthase-like protein	2.78	Yes
lmo2454	hypothetical protein	2.78	Yes
dltA	D-alanine--poly(phosphoribitol) ligase subunit 1	2.77	Yes
lmo1690	YfhP protein	2.76	Yes
lmo0359	fructose-bisphosphate aldolase	2.75	Yes
lmo1201	uroporphyrin-III C-methyltransferase/uroporphyrinogen-III synthase	2.75	Yes
ctaB	protoheme IX farnesyltransferase	2.75	Yes
lmo1417	BtlA/MFS transporter	2.72	Yes
lmo1153	glycerol dehydratase	2.71	Yes
lmo0818	cation-transporting ATPase PacL	2.69	Yes
lmo0539	tagatose 1,6-diphosphate aldolase	2.69	Yes
lmo0445	HTH domain-containing protein	2.69	Yes
lmo1225	MarR family transcriptional regulator	2.68	Yes
glnA	glutamine synthetase	2.68	Yes
lmo0217	cell division protein DivIC	2.68	Yes
lmo0400	PTS system fructose-specific IIC component	2.65	Yes
lmo1172	response regulator NasT	2.65	Yes
lmo0540	penicillin-binding protein	2.62	Yes
lmo2695	dihydroxyacetone kinase subunit DhaK	2.60	Yes
lmo0111	EAL domain protein	2.60	Yes
lmo1161	ethanolamine utilization protein	2.60	Yes
lmo2374	aspartate kinase	2.59	Yes
lmo2349	amino acid-binding protein/ ABC transporter	2.59	Yes
lmo1694	NAD-dependent epimerase/dehydratase family protein	2.58	Yes
lmo0953	hypothetical protein	2.57	Yes
miaA	tRNA delta(2)-isopentenylpyrophosphate transferase	2.57	Yes

pyrP	uracil-xanthine permease	2.57	Yes
lmo0909	GntR family transcriptional regulator	2.55	Yes
leuC	isopropylmalate isomerase large subunit	2.54	Yes
lmo0456	cytosine/purines/uracil/thiamine/allantoin permease family protein	2.53	Yes
lmo2087	MATE efflux family protein	2.53	Yes
ispG	4-hydroxy-3-methylbut-2-en-1-yl diphosphate synthase	2.53	Yes
cbiQ	cobalt transport protein	2.51	Yes
lmo2343	monooxygenase	2.51	Yes
lmo2130	amino acid-polyamine-organocation transporter	2.50	Yes
lmo2269	yhzC protein	2.50	Yes
lmo0959	llm protein	2.49	Yes
lmo0331	cell wall surface anchor family protein	2.49	Yes
lmo1056	hypothetical protein	2.48	Yes
lmo0529	glycosyl transferase	2.45	Yes
qoxB	cytochrome c quinol oxidase	2.45	Yes
lmo1247	hypothetical protein	2.44	Yes
lmo1830	short chain dehydrogenase	2.43	Yes
lmo1880	ribonuclease HI	2.43	Yes
lmo1375	peptidase T	2.42	Yes
lmo2515	transcriptional regulatory protein DegU	2.42	Yes
lmo1467	phosphate starvation-inducible protein PhoH	2.41	Yes
lmo1883	chitinase	2.41	Yes
lmo0793	uncharacterized membrane protein ydfK	2.41	Yes
lmo0387	hypothetical protein	2.40	Yes
lmo0342	transketolase	2.40	Yes
lmo2794	nucleoid occlusion protein	2.39	Yes
lmo0849	amidase	2.39	Yes
lmo0170	hypothetical protein	2.39	Yes
lmo2698	phosphosugar-binding transcriptional regulator	2.39	Yes
kdpC	potassium-transporting atpase c chain	2.38	Yes
lmo0627	pepdidoglycan bound protein	2.37	Yes
lmo1112	hypothetical protein	2.36	Yes
lmo1718	hypothetical protein	2.35	Yes
lmo0655	serine/threonine protein phosphatase	2.35	Yes
lmo0956	N-acetylglucosamine-6-phosphate deacetylase	2.35	Yes
bvrB	PTS system beta-glucoside-specific phosphotransferase enzyme II ABC component	2.33	Yes
fliH	flagellar assembly protein H	2.32	Yes
lmo1720	PTS system cellobiose-specific IIB component	2.32	Yes
lmo1070	hypothetical protein	2.32	Yes

lmo2387	hypothetical protein	2.30	Yes
lmo2431	iron complex transport system substrate-binding protein	2.30	Yes
lmo0560	glutamate dehydrogenase	2.29	Yes
lmo0984	LytTr DNA-binding domain family	2.27	Yes
lmo0819	hypothetical protein	2.25	Yes
lmo0066	hypothetical protein	2.25	Yes
hisD	histidinol dehydrogenase	2.24	Yes
lmo0994	hypothetical protein	2.24	Yes
lmo0905	hypothetical protein	2.23	Yes
lmo0760	carboxylesterase	2.23	Yes
zurA	zinc transport system ATP-binding protein	2.23	Yes
lmo0904	hypothetical protein	2.23	Yes
ilvA	threonine dehydratase	2.23	Yes
lmo2022	aminotransferase	2.22	Yes
lmo0732	peptidoglycan binding protein	2.22	Yes
lmo0458	hydantoinase/oxoprolinase	2.22	Yes
lmo1216	N-acetylmuramoyl-L-alanine amidase	2.22	Yes
lmo1665	ureI protein	2.22	Yes
lmo2750	para-aminobenzoate synthase	2.20	Yes
lmo0104	hypothetical protein	2.19	Yes
lmo0605	mate family multi antimicrobial extrusion protein	2.18	Yes
lmo1140	glyoxalase	2.18	Yes
lmo0618	protein kinase domain-containing protein	2.18	Yes
lmo2464	TetR family transcriptional regulator	2.17	Yes
lmo1416	hypothetical protein	2.17	Yes
lmo0820	acetyltransferase	2.15	Yes
lmo1369	phosphate acetyl/butyryltransferase	2.14	Yes
lmo1652	hypothetical protein	2.13	Yes
lmo1884	xanthine permease	2.13	Yes
lmo2852	Anhydro-N-acetylmuramic acid kinase	2.12	Yes
lmo0269	hypothetical protein	2.10	Yes
recU	recombination protein U	2.10	Yes
lmo2243	methylphosphotriester-DNA alkyltransferase	2.10	Yes
lmo0729	A disintegrin and metalloproteinase with thrombospondin motifs 2	2.10	Yes
hisE	phosphoribosyl-ATP pyrophosphatase	2.09	Yes
lmo1156	glycerol dehydratase reactivation factor large subunit	2.08	Yes
AckA2	acetate kinase 2	2.07	Yes
hisB	imidazoleglycerol-phosphate dehydratase	2.07	Yes
ltrC	low temperature requirement C protein	2.07	Yes

lmo0608	ABC transporter	2.06	Yes
lmo0822	MerR family transcriptional regulator	2.05	Yes
lmo2743	putative translaldolase	2.05	Yes
lmo0435	peptidoglycan binding protein	2.05	Yes
lmo2416	lipoprotein	2.05	Yes
ilvC	ketol-acid reductoisomerase	2.04	Yes
lmo2690	TetR family transcriptional regulator	2.04	Yes
lmo0649	GntR family transcriptional regulator	2.03	Yes
lmo1943	hypothetical protein	2.03	Yes
cobD	threonine-phosphate decarboxylase	2.02	Yes
lmo0551	hypothetical protein	2.02	Yes
lmo0812	HD domain-containing protein	2.02	Yes
lmo2056	uncharacterized membrane protein ylbC	2.01	Yes
lmo1719	PTS system cellobiose-specific IIA component	2.01	Yes
cbiP	cobyric acid synthase	2.01	Yes
lmo0717	transglycosylase	2.01	Yes
