# Supplementary material for: Deletion of glutaredoxin promotes oxidative tolerance and intracellular infection in Listeria monocytogenes
Source: Virulence. 2019 Nov 2;10(1):910–24. doi: 10.1080/21505594.2019.1685640 (PMC6844310; doi:10.1080/21505594.2019.1685640)
Supplement: Supplemental Material [file kvir-10-01-1685640-s001.zip › Grx supporting Table S3.pdf]

Table S3. Genes identified by transcriptome analysis as significantly down-regulated in *L. monocytogenes* Δ*grx* mutant.

Gene name	Annotation	Fold change (EGD-e /Δ*grx*)	Significance
lmo2827	MarR family transcriptional regulator	∞	Yes
lmo2327	hypothetical protein	∞	Yes
lmo2857	uncharacterized protein ynaF	∞	Yes
lmo2328	putative transcriptional regulator	∞	Yes
lmo2271	hypothetical protein	∞	Yes
lmo2326	protein gp41	∞	Yes
lmo0780	hypothetical protein	∞	Yes
lmo0338	Inner capsid protein lambda-1	∞	Yes
lmo0478	secreted hypothetical protein	∞	Yes
lmo0150	hypothetical protein	∞	Yes
lmo0631	PTS system fructose-specific IIA component	∞	Yes
lmo1613	hypothetical protein	∞	Yes
lmo2093	hypothetical protein	∞	Yes
lmo2764	glucokinase	126.32	Yes
lmo0051	accessory gene regulator protein A	103.00	Yes
lmo2763	PTS system cellobiose-specific IIC component	76.48	Yes
lmo0736	ribose-5-phosphate isomerase B	73.17	Yes
lmo0735	ribulose-phosphate 3 epimerase superfamily	67.92	Yes
lmo0050	accessory gene regulator protein C	62.97	Yes
lmo0128	phage holin	56.29	Yes
lmo0126	hypothetical protein	55.49	Yes
lmo0737	Elongation factor	55.02	Yes
lmo0738	PTS system beta-glucosides-specific IIA	54.32	Yes
lmo0048	accessory gene regulator protein	50.96	Yes
lmo0119	hypothetical protein	50.76	Yes
lmo0180	sugar ABC transporter	46.58	Yes
lmo0739	glycosyl hydrolase	39.01	Yes
lmo0477	secreted hypothetical protein	36.95	Yes
lmo0124	hypothetical protein	36.23	Yes
lmo0123	minor structural protein	34.89	Yes
lmo0181	sugar ABC transporter	33.92	Yes
lmaB	antigen B	31.76	Yes
lmo2801	N-acetylmannosamine-6-phosphate 2-epimerase	30.81	Yes
lmo0121	hypothetical protein	30.22	Yes
lmo0122	hypothetical protein	29.56	Yes
lmo0129	N-acetylmuramoyl-L-alanine amidase	29.34	Yes
lmo0125	hypothetical protein	28.03	Yes
lmo0179	sugar ABC transporter permease	27.95	Yes

lmo2324	hypothetical protein	25.04	Yes
lmo2799	PTS system mannitol-specific IIB component	22.34	Yes
lmaC	antigen C	22.29	Yes
lmo1999	glucosamine-fructose-6-phosphate aminotransferase	21.88	Yes
lmo2761	glycosyl hydrolase	21.84	Yes
lmaA	antigen A	20.75	Yes
lmo2000	PTS system mannose-specific IID component	20.56	Yes
lmo2644	hypothetical protein	20.01	Yes
lmo1998	fructoselysine 6-phosphate deglycase	19.79	Yes
lmo2800	myo-inositol 2-dehydrogenase	19.78	Yes
lmo2646	hypothetical protein	19.46	Yes
lmo0127	hypothetical protein	18.78	Yes
lmaD	hypothetical protein	18.51	Yes
lmo0120	hypothetical protein	17.82	Yes
lmo2647	creatinine amidohydrolase	16.74	Yes
lmo2121	maltose phosphorylase	15.77	Yes
lmo0412	hypothetical protein	15.62	Yes
lmo2122	maltodextrose utilization membrane protein	15.54	Yes
lmo2762	PTS system cellobiose-specific IIB component	14.19	Yes
lmo2316	DNA adenine methylase	13.31	Yes
lmo1997	deoxyribonucleoside regulator	13.01	Yes
lmo2123	maltose/maltodextrin ABC transporter permease	12.84	Yes
lmo1653	cellsurface protein	12.42	Yes
lmo2001	PTS system mannose-specific IIC component	12.35	Yes
lmo2317	replication initiation and membrane attachment protein	12.28	Yes
lmo2798	hydrolase	12.19	Yes
lmo2306	hypothetical protein	11.73	Yes
lmo0182	alpha-D-xyloside xylohydrolase	11.55	Yes
lmo2648	phosphotriesterase family protein	11.17	Yes
lmo2308	single-strand binding protein subfamily	10.50	Yes
lmo2318	hypothetical protein	10.20	Yes
lmo2323	hypothetical protein	10.19	Yes
lmo2303	hypothetical protein	10.18	Yes
lmo0178	xylose repressor protein	8.88	Yes
ulaA	PTS system ascorbate-specific IIC component	8.72	Yes
lmo2002	PTS system mannose-specific IIB component	8.63	Yes
lmo2292	hypothetical protein	8.42	Yes
lmo2669	hypothetical protein	8.26	Yes
argC	N-acetyl-gamma-glutamyl-phosphate reductase	8.24	Yes
argJ	bifunctional ornithine	8.15	Yes

	acetyltransferase/N-acetylglutamate synthase		
argG	argininosuccinate synthase	8.15	Yes
lmo2282	hypothetical protein	8.14	Yes
lmo0480	TetR family transcriptional regulator	7.87	Yes
lmo2291	major tail shaft protein	7.51	Yes
lmo0813	fructokinase	7.38	Yes
lmo2337	DeoR family transcriptional regulator	7.20	Yes
groES	co-chaperonin	7.19	Yes
lmo2650	PTS system ascorbate-specific IIB component	7.03	Yes
lmo0183	alpha-glucosidase	6.81	Yes
lmo2295	hypothetical protein	6.52	Yes
fruB	fructose-1-phosphate kinase	6.43	Yes
lmo2223	hypothetical protein	6.23	Yes
lmo2439	hypothetical protein	6.08	Yes
lmo2797	PTS system mannitol-specific IIA component	5.91	Yes
lmo2294	hypothetical protein	5.87	Yes
lmo2819	aminoacylase	5.85	Yes
lmo2299	portal protein	5.82	Yes
lmo2286	hypothetical protein	5.76	Yes
lmo2293	hypothetical protein	5.75	Yes
lmo2296	hypothetical protein	5.66	Yes
lmo0423	RNA polymerase factor sigma C	5.61	Yes
lmo1717	hypothetical protein	5.61	Yes
lmo2126	neopullulanase	5.60	Yes
lmo2300	terminase large subunit from bacteriophage A118	5.55	Yes
lmo1776	hypothetical protein	5.36	Yes
lmo0303	lysin rich protein	5.31	Yes
lmo2288	hypothetical protein	5.11	Yes
lmo2298	hypothetical protein	5.08	Yes
lmo2283	hypothetical protein	5.04	Yes
lmo2651	PTS system ascorbate-specific IIA component	4.97	Yes
lmo2124	maltose/maltodextrin ABC transporter	4.96	Yes
lmo2287	methyl-accepting chemotaxis protein	4.75	Yes
argH	argininosuccinate lyase	4.71	Yes
lmo2829	nitroreductase	4.62	Yes
lmo1227	uracil-DNA glycosylase	4.57	Yes
lmo0903	OsmC/Ohr family protein	4.56	Yes
lysA	L-alanoyl-D-glutamate peptidase	4.54	Yes
lmo1069	hypothetical protein	4.52	Yes
lmo1028	putative transcriptional regulator	4.52	Yes
alsS	acetolactate synthase	4.50	Yes
lmo2285	hypothetical protein	4.47	Yes

lmo1138	ATP-dependent Clp protease proteolytic subunit	4.39	Yes
lmo2284	hypothetical protein	4.38	Yes
lmo0184	oligo-1,6-glucosidase	4.34	Yes
eutD	phosphotransacetylase	4.33	Yes
lmo0197	regulatory protein SpoVG	4.33	Yes
lmo1992	alpha-acetolactate decarboxylase	4.13	Yes
lmo2297	scaffolding protein	4.07	Yes
mscL	large-conductance mechanosensitive channel protein	4.06	Yes
lmo0791	lipoprotein	3.98	Yes
lmo0770	GntR family transcriptional regulator	3.96	Yes
lmo2054	hypothetical protein	3.95	Yes
lmo2289	hypothetical protein	3.94	Yes
lmo0641	cadmium-translocating P-type ATPase	3.90	Yes
lmo2581	putative ABC transport system permease protein	3.87	Yes
lmo2290	hypothetical protein	3.73	Yes
groEL	60 kDa chaperonin	3.73	Yes
lmo2301	phage terminase small subunit	3.71	Yes
lmo2003	GntR family transcriptional regulator	3.66	Yes
lmo0547	deoxyribonucleoside regulator	3.65	Yes
kat	catalase	3.57	Yes
sigB	RNA polymerase sigma factor SigB	3.55	Yes
lmo1604	Thioredoxin-like protein ykuU	3.53	Yes
lmo2125	maltose/maltodextrin ABC transporter maltose/maltodextrin-binding protein	3.52	Yes
lmo1802	DNA-binding protein	3.46	Yes
panC	pantoate--beta-alanine ligase	3.45	Yes
lmo2661	ribulose-phosphate 3-epimerase family protein	3.41	Yes
lmo2144	GntR family transcriptional regulator	3.40	Yes
lmo2114	putative ABC transport system ATP-binding protein	3.39	Yes
lmo0919	ABC transporter ATP-binding protein	3.39	Yes
lmo2662	ribose 5-phosphate isomerase B	3.38	Yes
lmo0536	Glycoside hydrolase	3.36	Yes
lmo2580	putative ABC transport system ATP-binding protein	3.35	Yes
lmo0918	PRD/PTS system IIA 2 domain-containing protein	3.32	Yes
panD	aspartate alpha-decarboxylase	3.31	Yes
lmo2728	MerR family transcriptional regulator	3.28	Yes
lstR	lineage-specific thermal regulator protein	3.27	Yes
lmo0278	sugar ABC transporter	3.25	Yes
lmo1409	multidrug resistance transporter	3.25	Yes
lmo2707	hypothetical protein	3.24	Yes
lmo2818	major facilitator family transporter	3.19	Yes

mpl	Zinc metalloproteinase precursor	3.18	Yes
rsbX	serine phosphatase	3.18	Yes
lmo1249	Arginine-tRNA ligase	3.17	Yes
lmo2010	DNA-binding response regulator	3.13	Yes
lmo2361	Putative HTH-type transcriptional regulator YwgB	3.13	Yes
fruA	PTS system fructose-specific IIA component	3.13	Yes
lmo0800	uncharacterized protein yqkB	3.07	Yes
lmo0815	MarR family transcriptional regulator	3.06	Yes
lmo2273	hypothetical protein	3.05	Yes
hrcA	heat-inducible transcription repressor HrcA	3.05	Yes
arpJ	amino acid ABC transporter	3.03	Yes
lmo2828	hypothetical protein	3.01	Yes
lmo0917	beta-glucosidase	2.99	Yes
grpE	heat shock protein GrpE	2.96	Yes
lmo1211	hypothetical protein	2.96	Yes
inlC	internalin C	2.95	Yes
lmo0421	FtsW/RodA/SpoVE family protein	2.93	Yes
lmo2331	hypothetical protein	2.89	Yes
lmo0471	hypothetical protein	2.89	Yes
lmo1710	flavodoxin	2.87	Yes
lmo1008	hypothetical protein	2.86	Yes
flaA	flagellin	2.85	Yes
lmo2113	heme peroxidase	2.85	Yes
lmo0081	hypothetical protein	2.85	Yes
lmo0786	FMN-dependent NADH-azoreductase 2	2.84	Yes
lmo0070	hypothetical protein	2.81	Yes
lmo0613	zinc-dependent alcohol dehydrogenase	2.81	Yes
lmo2251	polar amino acid transport system ATP-binding protein	2.78	Yes
lmo0113	Isoleucine-tRNA ligase	2.78	Yes
lmo1306	hypothetical protein	2.77	Yes
lmo2415	SUF system FeS assembly ATPase	2.77	Yes
int	integrase	2.74	Yes
lmo2585	hypothetical protein	2.72	Yes
lmo2579	hypothetical protein	2.71	Yes
lmo2584	formate dehydrogenase accessory protein	2.71	Yes
lmo2796	glucokinase	2.71	Yes
lmo2676	ImpB/MucB/SamB family protein	2.71	Yes
cysS	cysteinyl-tRNA synthetase	2.71	Yes
lmo2275	hypothetical protein	2.70	Yes
lmo1003	phosphotransferase system enzyme I	2.70	Yes
lmo0041	phosphosugar-binding protein	2.69	Yes

cysK	cysteine synthase A	2.69	Yes
rplS	50S ribosomal protein L19	2.62	Yes
lmo2637	lipoprotein	2.62	Yes
lmo2102	glutamine amidotransferase subunit PdxT	2.61	Yes
lmo1400	acetyltransferase	2.60	Yes
lmo0047	lipoprotein	2.60	Yes
tagB	CDP-glycerol:glycerophosphate glycerophosphotransferase	2.59	Yes
lmo0802	RelA/SpoT domain-containing protein	2.59	Yes
lmo2177	uncharacterized protein yvlA	2.58	Yes
lmo1050	FMN-binding split barrel domain-containing protein	2.56	Yes
lmo2230	arsenate reductase	2.56	Yes
lmo0502	arabinose-5-phosphate isomerase	2.55	Yes
lmo0309	hypothetical protein	2.54	Yes
lmo1118	hypothetical protein	2.52	Yes
clpB	ATP-dependent Clp protease ATP-binding subunit ClpB	2.51	Yes
lmo0056	small heat shock protein of Clostridium acetobutylicum	2.51	Yes
panB	3-methyl-2-oxobutanoate hydroxymethyltransferase	2.48	Yes
lmo2274	hypothetical protein	2.47	Yes
lmo0208	hypothetical protein	2.47	Yes
plcB	phospholipase	2.46	Yes
lmo0617	lipoprotein	2.44	Yes
lmo2705	hypothetical protein	2.44	Yes
acpD	azoreductase	2.41	Yes
lmo1752	Coiled-coil domain-containing protein 138	2.39	Yes
lmo2115	putative ABC transport system permease protein	2.39	Yes
lmo2111	nitroreductase	2.39	Yes
lmo2701	uncharacterized protein yaaL	2.39	Yes
lmo2843	hypothetical protein	2.38	Yes
lmo2027	internalin	2.37	Yes
lmo2591	GW repeat-containing surface protein	2.36	Yes
lmo0789	phenazine biosynthesis protein	2.35	Yes
lmo0341	pediocin immunity protein	2.35	Yes
lmo0932	uncharacterized membrane protein yhjE	2.35	Yes
lmo1066	inositol monophosphatase family protein	2.34	Yes
lmo0095	hypothetical protein	2.32	Yes
ctc	50S ribosomal protein L25/general stress Ctc	2.32	Yes
lmo2340	indigoidine synthase A-like protein	2.32	Yes
lmo2168	lactoylglutathione lyase	2.31	Yes
lmo0304	hypothetical protein	2.31	Yes

lmo1861	hypothetical protein	2.31	Yes
lmo1078	UTP--glucose-1-phosphate uridylyltransferase	2.30	Yes
lmo0731	hypothetical protein	2.28	Yes
lmo0859	sugar ABC transporter	2.26	Yes
lmo0131	EAL domain-containing protein	2.24	Yes
lmo2817	thermostable carboxypeptidase 1	2.23	Yes
lmo2365	RofA regulatory protein	2.23	Yes
lmo2110	mannose-6-phosphate isomerase	2.23	Yes
lmo1862	lipase/acylhydrolase	2.23	Yes
lmo1412	modulates DNA topology	2.23	Yes
lmo0643	transaldolase	2.23	Yes
lmo0230	hypothetical protein	2.22	Yes
lmo0790	putative transcription regulator	2.22	Yes
guaB	Inosine-5'-monophosphate dehydrogenase	2.21	Yes
lmo0799	blue-light photoreceptor	2.21	Yes
lmo2675	hypothetical protein	2.20	Yes
lmo1255	PTS system trehalose-specific IIB component	2.19	Yes
lmo1274	DNA protecting protein DprA	2.18	Yes
ldh	L-lactate dehydrogenase	2.18	Yes
rplM	50S ribosomal protein L13	2.17	Yes
lmo2729	4-carboxymuconolactone decarboxylase	2.17	Yes
lmo2816	major facilitator family transporter	2.17	Yes
cinA	competence damage-inducible protein A	2.17	Yes
lmo0983	glutathione peroxidase	2.17	Yes
lmo1975	DNA polymerase IV	2.15	Yes
lmo0209	hypothetical protein	2.14	Yes
actA	actin-assembly inducing protein precursor	2.14	Yes
lmo2166	cyclic nucleotide-binding protein	2.12	Yes
lmo1289	internalin-like protein	2.11	Yes
pfkA	6-phosphofructokinase	2.08	Yes
lmo0042	membrane-associated protein	2.08	Yes
lmo1709	methionine aminopeptidase	2.08	Yes
lmo2101	pyridoxal biosynthesis lyase PdxS	2.07	Yes
lmo1825	bifunctional phosphopantothenoylcysteine decarboxylase/phosphopantothenate synthase	2.06	Yes
iap	invasion associated secreted endopeptidase	2.06	Yes
trpS	tryptophanyl-tRNA synthetase	2.05	Yes
lmo0030	hydrolase/haloacid dehalogenase-like family	2.03	Yes
lmo1842	methyl-accepting chemotaxis protein	2.01	Yes
lmo1220	MarR family transcriptional regulator	2.01	Yes
dnaK	molecular chaperone DnaK	2.00	Yes
