# Supplementary material for: Deletion of glutaredoxin promotes oxidative tolerance and intracellular infection in Listeria monocytogenes
Source: Virulence. 2019 Nov 2;10(1):910–24. doi: 10.1080/21505594.2019.1685640 (PMC6844310; doi:10.1080/21505594.2019.1685640)
Supplement: Supplemental Material [file kvir-10-01-1685640-s001.zip › Grx supporting Table S4.pdf]

Table S4. Cell lines used for the study of the InlA- and InlB-invasion pathways (Pizarro-Cerda et al., 2012).

Cell line	Species	Cell type	Receptors
Caco-2	Human	Colorectal adenocarcinoma	E-cadherin and Met
CHO	Hamster	Ovary	Met
GPC16	Guinea pig	Epithelial X	E-cadherin and Met
Hela	Human	Cervix carcinoma	Met
Hep-2	Human	Larynx carcinoma	Met
HepG2	Human	Hepatocarcinoma	Met
JEG-3	Human	Trophoblasts	E-cadherin and Met
L2071hEcad	Mouse	Fibroblasts	E-cadherin and Met
LoVo	Human	Colorectal carcinoma	E-cadherin and Met
REF-52	Rat	Embryonic fibroblasts	Met
TIB73	Mouse	Embryonic hepatocyte	Met
Vero	African green monkey	Kidney	Met
