# Supplementary figures and images for: Deletion of glutaredoxin promotes oxidative tolerance and intracellular infection in Listeria monocytogenes
Source: Virulence. 2019 Nov 2;10(1):910–24. doi: 10.1080/21505594.2019.1685640 (PMC6844310; doi:10.1080/21505594.2019.1685640)

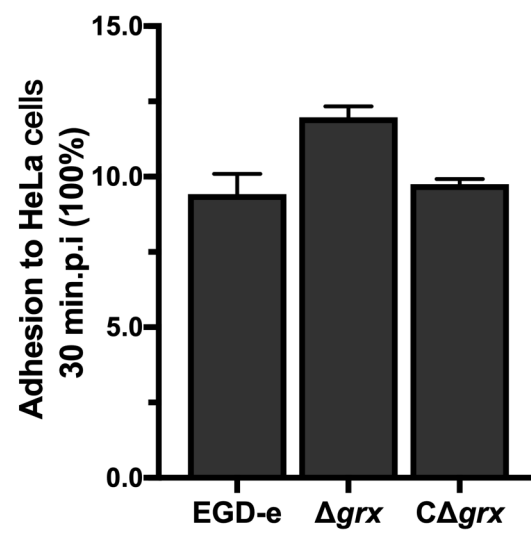

**Figure S2.** Adhesion ability of *L. monocytogenes* strains to HeLa cells.

Supplement: Supplemental Material [file kvir-10-01-1685640-s001.zip › Grx supporting Figure S2.pdf]
